# CRISPR/Cas in food microbial biotechnology: from genome editing to precision microbiology

**DOI:** 10.3389/fmicb.2026.1996037

**Published:** 2026-10-30

**Authors:** Edyta Juszczuk-Kubiak, Natalia Piłka, Ewelina Zielińska, Marek Roszko

**Affiliations:** 1 Laboratory of Genetic Engineering, Department of Biotechnology, Prof. Wacław Dąbrowski Institute of Agricultural and Food Biotechnology-State Research Institute, Warsaw, Poland; 2 Department of Food Safety and Chemical Analysis, Prof. Wacław Dąbrowski Institute of Agricultural and Food Biotechnology-State Research Institute, Warsaw, Poland

**Keywords:** biosafety, CRISPR/Cas tools, food biotechnology, food safety, metabolic engineering, microbial genome editing, regulatory frameworks

## Abstract

CRISPR/Cas technologies have opened new opportunities for the precise genetic engineering of microorganisms relevant to food biotechnology. Their programmability enables targeted genome modification, transcriptional regulation, sequence-specific antimicrobial activity, and nucleic acid detection, offering new strategies to improve fermentation processes, microbial safety, and the functional properties of food-associated strains. However, experimental evidence varies considerably across microorganisms and applications, and successful genome editing does not necessarily translate into improved technological performance. This review examines recent advances in CRISPR/Cas-based engineering of starter cultures, probiotic strains, and production microorganisms, alongside emerging strategies for bacteriophage resistance, pathogen and biofilm control, and food diagnostics. Particular emphasis is placed on distinguishing applications experimentally validated in food-related settings from proof-of-concept studies conducted in laboratory or biomedical contexts. The review also evaluates the major barriers to industrial implementation, including strain-specific editing efficiency, genetic stability, process robustness, and validation in food matrices. It also considers the distinct biosafety and regulatory implications of production microorganisms removed or inactivated during processing versus viable engineered cultures retained in final products. By integrating advances in molecular engineering with evidence of functional performance and practical implementation requirements, this review highlights both the opportunities and current limitations of CRISPR/Cas technologies in food microbial biotechnology.

## 1 Introduction

CRISPR/Cas (Clustered Regularly Interspaced Short Palindromic Repeats/CRISPR-associated proteins) systems are derived from adaptive immune mechanisms that protect bacteria and archaea against invading genetic elements, particularly bacteriophages (Barrangou et al., 2007; Marraffini and Sontheimer, 2010). The demonstration that Cas nucleases can be programmed to target specific nucleic acid sequences enabled their repurposing for targeted genome engineering (Jinek et al., 2012; Doudna and Charpentier, 2014). Subsequent development has produced a diverse technological platform that includes alternative Cas effectors, CRISPR interference (CRISPRi), and increasingly efficient strategies for genome modification and transcriptional regulation (Qi et al., 2013; Pickar-Oliver and Gersbach, 2019; Ding et al., 2020). As a result, CRISPR-based technologies are now widely used in biomedical, agricultural, and biotechnological research (Doudna and Charpentier, 2014; Pickar-Oliver and Gersbach, 2019).

Microbial biotechnology is a key area for applying these tools (Stout et al., 2017; Ding et al., 2020). Microorganisms are fundamental to food fermentation and biopreservation, producing numerous metabolites that influence food quality, safety, sensory characteristics, and functional properties (Stout et al., 2017; Mougiakos et al., 2018). Conventional strain improvement relies primarily on screening, selection, adaptive evolution, or random mutagenesis. In contrast, CRISPR/Cas technologies enable targeted modification of defined genetic loci and can therefore support the rational development of microorganisms with specific technological or functional traits (Stout et al., 2017; Mougiakos et al., 2018). However, implementing these technologies in food-associated microorganisms is complicated by differences in genetic accessibility, transformation efficiency, restriction–modification and other endogenous defense systems, DNA repair mechanisms, and strain-specific physiology (Mougiakos et al., 2018; Leenay et al., 2019; Mu et al., 2022; Cui and Qu, 2024). Editing protocols developed for one microorganism may therefore require extensive optimization before they can be applied to another species or even to another strain of the same species (Leenay et al., 2019; Mu et al., 2022; Cui and Qu, 2024).

Despite this broad potential, most CRISPR-engineered food microorganisms remain at the laboratory or proof-of-concept stage. Editing efficiency often depends on the microbial species, strain, genetic target, delivery method, and available DNA repair pathways. Moreover, successful genome modification does not by itself establish the industrial suitability of an engineered strain. Practical implementation requires genetic and phenotypic stability, reproducible fermentation performance, robustness under food-processing and storage conditions, and confirmation that the modification does not adversely affect product quality or safety (Mougiakos et al., 2018; Leenay et al., 2019; Mu et al., 2022; Cui and Qu, 2024; Xin et al., 2025). Food-grade and preferably marker-free construction strategies are also desirable, although the absence of selectable markers or persistent editing plasmids does not eliminate the need for molecular and phenotypic safety assessment (EFSA GMO Panel, 2011; Plavec and Berlec, 2020). The required assessment additionally depends on the intended use and resulting exposure. Production organisms removed or inactivated during processing present a different exposure scenario than viable starter cultures or probiotics that remain in the final food product (EFSA GMO Panel, 2011; Plavec and Berlec, 2020).

This review critically examines recent advances in CRISPR/Cas-based engineering of food-associated microorganisms, focusing on applications in microbial strain improvement, food bioprocessing, and food safety. It also discusses major limitations affecting editing efficiency and industrial translation, along with relevant biosafety, regulatory, and consumer considerations. Finally, it considers emerging directions and evaluates how CRISPR/Cas technologies may contribute to developing more precise, safer, and functionally tailored microbial systems for future food biotechnology.

## 2 CRISPR/Cas systems: principles and genome-editing strategies

CRISPR/Cas systems are adaptive immune mechanisms that protect bacteria and archaea against invading genetic elements, particularly bacteriophages and plasmids (Hille et al., 2018; Eliwa and Eldahshan, 2026). CRISPR arrays incorporate fragments of foreign nucleic acids and transcribe them into CRISPR RNAs (crRNAs), which guide Cas effectors to complementary targets during subsequent encounters (van der Oost et al., 2009; Koonin and Makarova, 2019). Class 1 systems use multi-protein effector complexes, whereas Class 2 systems rely on a single multi-domain effector; the latter architecture facilitated the adaptation of Cas9, Cas12, and Cas13 for biotechnology (Makarova et al., 2025; Wu et al., 2024; Singh et al., 2026). In engineered Cas9 systems, a single-guide RNA directs the nuclease to DNA adjacent to a protospacer-adjacent motif, producing a double-strand break, as illustrated in Figure 1 (Sternberg et al., 2014; Nishimasu et al., 2014). Cellular repair can then generate targeted deletions, insertions, substitutions, or replacements, often using homologous recombination coupled with CRISPR-mediated counter-selection (Barrangou and van Pijkeren, 2016; Jiang et al., 2013; Shaposhnikov et al., 2025).

**Figure 1:**
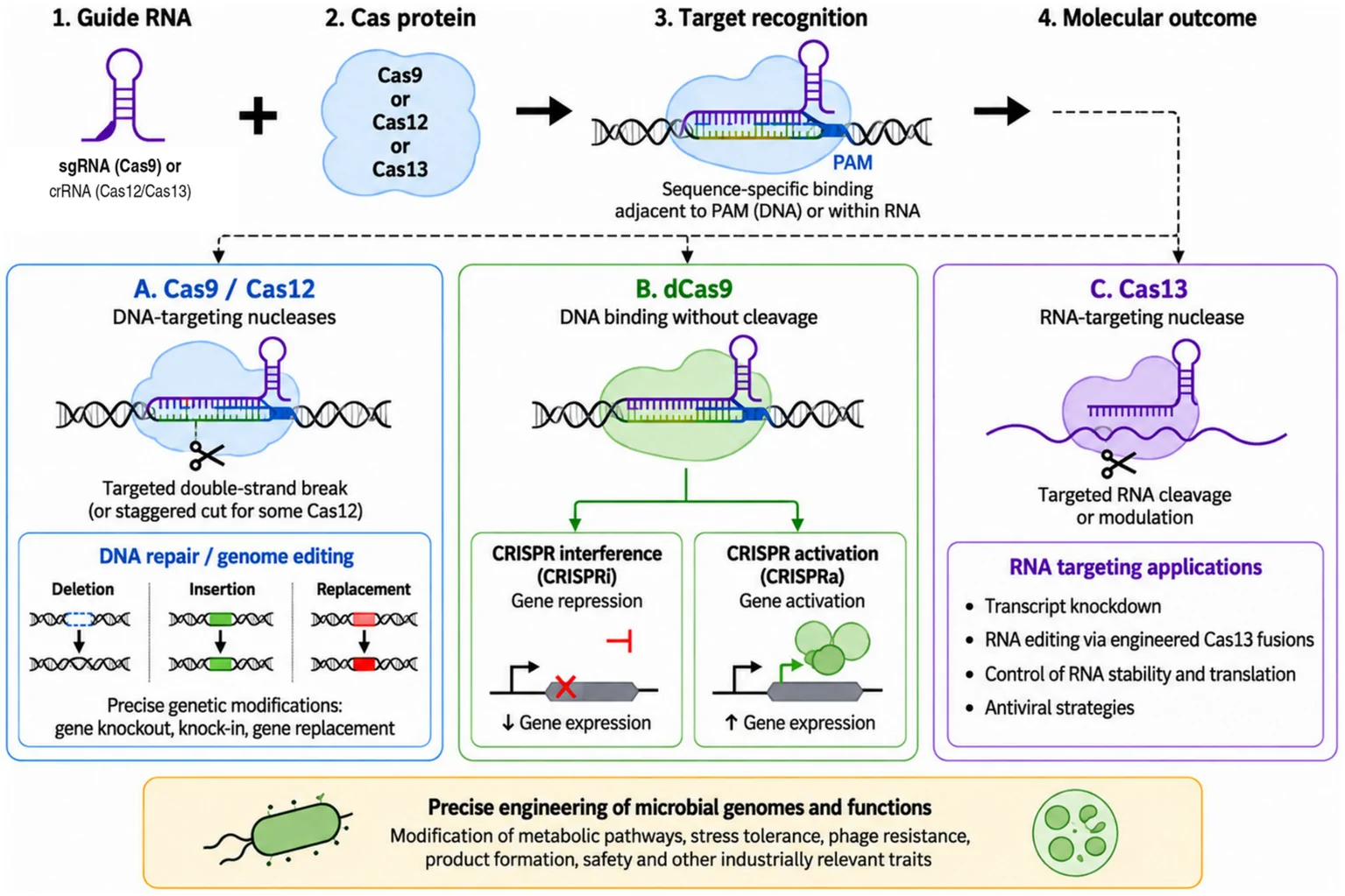
Conceptual schematic of major CRISPR/Cas-based strategies for microbial genome engineering. (A) Cas9 and Cas12 enable targeted DNA modification. (B) Catalytically inactive dCas9 supports transcriptional repression (CRISPRi) or activation (CRISPRa) without DNA cleavage. (C) Cas13 enables programmable RNA cleavage, whereas RNA editing requires engineered Cas13-based platforms. Cas, CRISPR-associated protein; CRISPR, clustered regularly interspaced short palindromic repeats; CRISPRa, CRISPR activation; CRISPRi, CRISPR interference; crRNA, CRISPR RNA; dCas9, catalytically inactive Cas9; DNA, deoxyribonucleic acid; PAM, protospacer-adjacent motif; RNA, ribonucleic acid; sgRNA, single-guide RNA; tracrRNA, trans-activating CRISPR RNA.

Cas12 and Cas13 broaden the available targeting range. Cas12 cleaves DNA but differs from Cas9 in PAM requirements and cleavage characteristics, whereas Cas13 recognizes RNA and supports sequence-specific transcript targeting (Abudayyeh et al., 2016; Wu et al., 2024; Saha et al., 2026). Catalytically inactive Cas proteins, particularly dCas9, enable programmable transcriptional repression (CRISPRi) or activation (CRISPRa) without DNA cleavage (Dominguez et al., 2016; Lv et al., 2023). Base editors enable defined nucleotide substitutions, whereas prime editing can introduce targeted substitutions and small insertions or deletions, generally without generating conventional double-strand DNA breaks (Liu D. et al., 2025; Li X. et al., 2026). Selection of an appropriate system depends on the host, intended modification, delivery efficiency, and DNA-repair capacity, which remain major constraints in genetically diverse food-associated microorganisms (Shaposhnikov et al., 2025; Singh et al., 2026).

## 3 CRISPR/Cas applications in food microbial biotechnology

Applications of CRISPR/Cas technologies in food microbial biotechnology can be broadly divided into two complementary areas: (1) the engineering of beneficial and industrial microorganisms and (2) the targeted control of microorganisms that compromise food quality or safety (Figure 2). The following sections examine both areas, with particular attention to the functional validation of engineered phenotypes and their potential translation into food-production environments.

**Figure 2:**
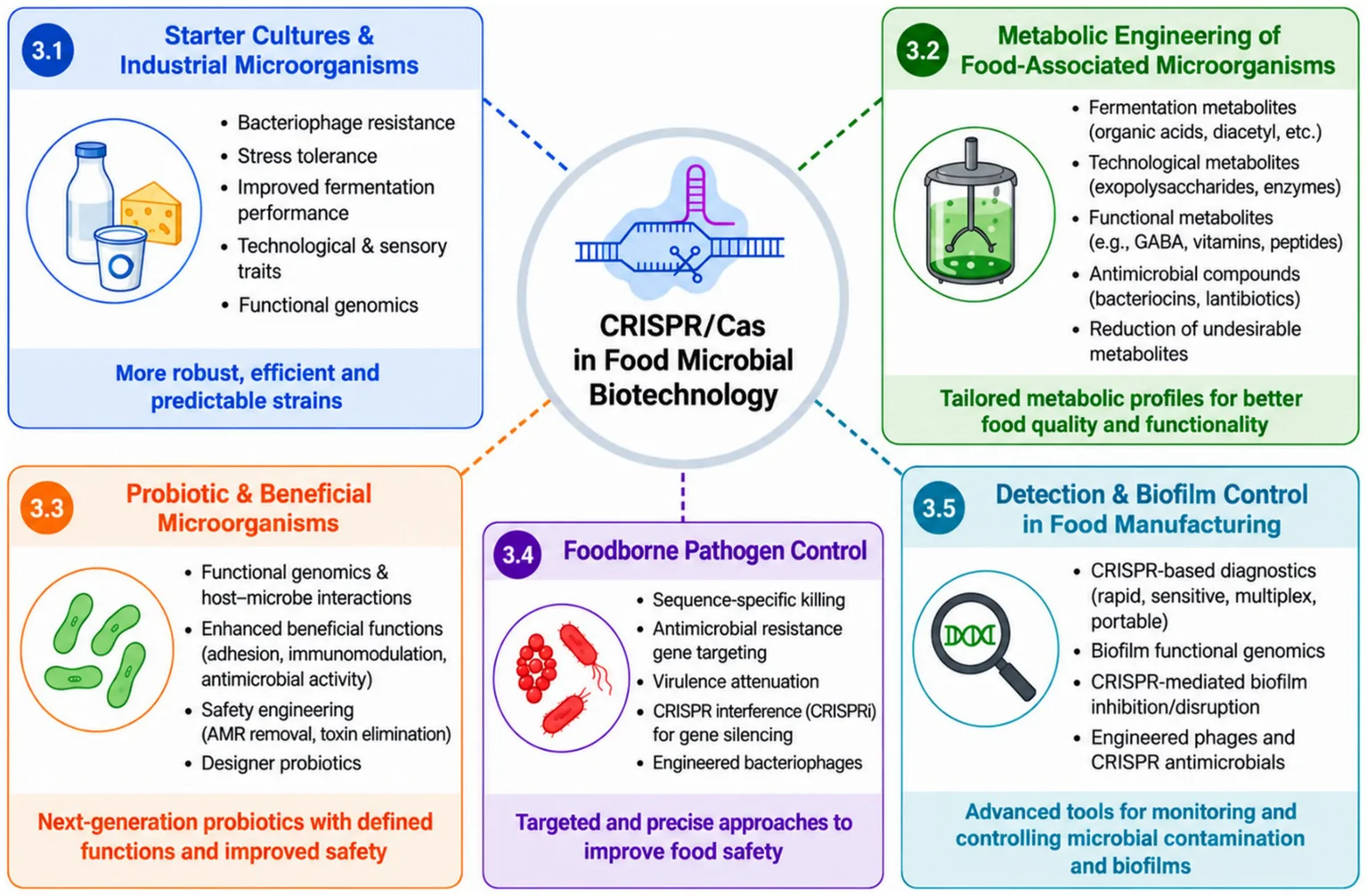
Conceptual schematic of major applications of CRISPR/Cas technologies in food-associated microorganisms. The central CRISPR/Cas platform enables targeted interventions across five key areas: (3.1) Starter culture and industrial strain improvement, (3.2) Metabolic engineering, (3.3) Probiotic and beneficial microorganism engineering, (3.4) Foodborne pathogen control, and (3.5) Pathogen detection and biofilm management in food manufacturing. AMR, antimicrobial resistance; Cas, CRISPR-associated protein; CRISPR, clustered regularly interspaced short palindromic repeats; GABA, γ-aminobutyric acid.

### 3.1 Engineering starter cultures and industrial microorganisms

Starter cultures are central to fermented-food production, where their metabolic activity determines fermentation rate, process reproducibility, product stability, and many sensory characteristics (Leroy and De Vuyst, 2004; Bintsis, 2018). CRISPR/Cas technologies offer an opportunity to move beyond conventional strain selection and random mutagenesis toward targeted modification of genetic determinants linked to industrially relevant phenotypes (Stout et al., 2017; Jolicoeur et al., 2022; Lee et al., 2022). This is particularly important for lactic acid bacteria (LAB), whose technological properties are often highly strain-dependent and whose genetic manipulation remains more challenging than that of standard laboratory organisms (Leenay et al., 2019; Shaposhnikov et al., 2025). In food-associated bacteria, the technological relevance of CRISPR/Cas initially stemmed from its natural role in defense against bacteriophages, while the subsequent development of programmable genome-editing systems has expanded its potential for the rational engineering of starter cultures and industrial strains (Barrangou and Horvath, 2012; Cui and Qu, 2024).

One of the earliest and most industrially relevant applications of CRISPR/Cas systems in starter cultures has been developing bacteriophage resistance (Barrangou et al., 2007; Barrangou and Horvath, 2012). Phage infection is a major challenge in industrial fermentation because disrupting starter-culture activity can delay fermentation or cause failure (Samson and Moineau, 2013; Mahony et al., 2017; Pujato et al., 2019). *Streptococcus thermophilus*, widely used in yoghurt and cheese manufacture, provided some of the earliest experimental evidence for CRISPR-mediated adaptive immunity. Deveau et al. (2008) analyzed 30 bacteriophage-insensitive mutants and showed that acquiring new spacers within the CRISPR1 locus was the predominant response to phage challenge. Iterative spacer acquisition increased overall phage resistance, whereas escape phages emerged through point mutations or deletions in targeted protospacer regions. These observations are directly relevant to starter-culture design because they demonstrate both the capacity of endogenous CRISPR systems to generate phage-resistant strains and the evolutionary pressure that may promote phage escape. This concept was subsequently demonstrated in industrial starter cultures. Mills et al. (2010) analyzed bacteriophage-insensitive mutants of industrial *S. thermophilus* starters and found that repeated phage challenge selected strains carrying one to four newly acquired spacers in CRISPR1, while one mutant additionally acquired a spacer in CRISPR3. Such findings established CRISPR-based phage immunization as a practical strategy for generating more robust dairy starter cultures. The relevance of endogenous CRISPR systems to dairy starter selection is further illustrated by Coban et al. (2024), who characterized the technological properties and CRISPR profiles of *Streptococcus thermophilus* isolates obtained from traditional yoghurt samples. This study highlights the value of combining technological characterization with CRISPR profiling to assess potential dairy starter cultures, although it did not involve CRISPR-mediated genome editing.

Beyond their natural role in phage defense and strain characterization, the endogenous CRISPR/Cas systems of starter bacteria can be repurposed as precise genome-engineering tools. In *S. thermophilus* LMD-9, Ma et al. (2024) exploited the endogenous CRISPR1 and CRISPR3 type II-A systems to introduce targeted deletions, nucleotide substitutions, and chromosomal insertions. Editing efficiency depended on the CRISPR locus and repair-template design; for example, a 785-bp *lacZ* deletion reached 90% efficiency using CRISPR3 with a 1.5-kb repair template, whereas other modifications, including nucleotide substitution, stop-codon introduction, and DNA insertion, achieved efficiencies of 75–100%. The system was further applied to six genes within the eps cluster, demonstrating its utility for functional analysis of technologically relevant pathways. These results illustrate an important transition from exploiting CRISPR as a natural defense mechanism to repurposing endogenous systems for rational engineering of starter-culture genomes.

CRISPR-based functional analysis can also reveal genetic determinants of technological properties that influence food quality (Kong et al., 2022a; Huang et al., 2025). In *S. thermophilus* S-3, Kong et al. (2022a) developed a CRISPR/nCas9 system enabling seamless single- and multigene editing and used it to investigate genes involved in exopolysaccharide (EPS) biosynthesis. The approach identified *epsA*, *epsB*, and *epsE* as key determinants of EPS formation, while deleting *epsC*, *epsE*, or *epsG* substantially altered EPS molecular weight, intrinsic viscosity, and monosaccharide composition. In particular, EPS molecular weight and intrinsic viscosity decreased by more than 2.5-fold and 19.8-fold, respectively, in the corresponding mutants. Because EPS structure contributes to the rheological and textural properties of fermented dairy products, these findings show how CRISPR-based functional genomics can identify genetic targets for future rational modification of starter-culture technological properties (Kong et al., 2022a). Importantly, this application should be distinguished from CRISPR-based metabolic engineering aimed specifically at increasing EPS production, which is discussed in Section 3.2.

Beyond phage resistance and characterizing technological traits, improving stress tolerance is another key objective in engineering industrial microorganisms. During fermentation, microorganisms may encounter temperature, osmotic, oxidative, acid, and product-associated stresses that impair cell growth and metabolic activity (Zhang et al., 2026). CRISPR-based engineering provides a way to identify and modify genetic determinants underlying cellular robustness. For example, Yang et al. (2023) used CRISPR/Cas9 to modify sterol biosynthesis in *Saccharomyces cerevisiae* by deleting *ERG5*, *ERG4*, and *ERG3*. The resulting triple mutant showed improved thermotolerance and produced 22.1 g/L ethanol at 37 °C, compared with 20.2 g/L in the wild-type strain; at 42 °C, ethanol production reached 9.4 g/L, compared with 3.3 g/L in the wild type. These results demonstrate that targeted modification of membrane sterol composition can translate increased stress tolerance into improved fermentation performance. CRISPR-derived base-editing technologies provide a complementary strategy for generating stress-tolerant phenotypic diversity without conventional gene deletion. Target-AID-mediated base editing of the general transcription factor gene *SPT15* in *S. cerevisiae* generated 36 variants with different responses to hyperosmotic, thermal, and ethanol stresses. Eighteen stress-tolerant mutants showed more than a 1.5-fold increase in fermentation capacity under the tested stress conditions, demonstrating the potential of targeted nucleotide diversification to engineer complex industrial phenotypes controlled by global transcriptional regulation (Liu Y. et al., 2021).

A particularly relevant example of CRISPR-mediated improvement under food-processing conditions was reported for the commercial baker’s yeast *S. cerevisiae* SPC-SNU 70-1. Cha et al. (2025) applied scarless CRISPR/Cas9 genome editing to improve fermentation performance in both low- and high-sugar doughs. In lean dough, deleting a gene involved in glucose repression enhanced preferential maltose utilization and leavening performance, whereas in sweet dough, increased glycerol biosynthesis and optimized cellular redox balance improved osmotic-stress tolerance and leavening. The glycerol-enhanced strain also showed improved freeze tolerance, and bread made with the engineered yeast showed improved storage properties without significant flavor changes. This study is particularly important because it shows that CRISPR-mediated modification of substrate utilization and stress-response pathways can translate into measurable technological benefits directly in a food matrix, not only under laboratory fermentation conditions.

CRISPR-based functional analysis has also clarified stress responses in LAB. In *Lacticaseibacillus paracasei*, editing of *glpF* and *glpO* confirmed their roles in glycerol metabolism and oxidative-stress tolerance and helped explain the greater resistance of cells from solid-state than liquid-state fermentation (Wu et al., 2025). This study established a genotype–phenotype relationship rather than directly producing a superior industrial strain, but identified targets for subsequent improvement. More broadly, evidence supports CRISPR applications in phage resistance, stress robustness, substrate utilization, and traits affecting fermentation and texture (Kong et al., 2022a; Yang et al., 2023; Cha et al., 2025; Wu et al., 2025). However, editing success or identification of a favorable allele does not demonstrate industrial superiority; engineered strains still require validation of genetic stability, fermentation kinetics, sensory compatibility, matrix robustness, and performance at scale (Cui and Qu, 2024; Shaposhnikov et al., 2025; Xin et al., 2025; Zhang et al., 2026).

Table 1 summarizes representative CRISPR/Cas-based strategies for improving starter cultures and industrial food microorganisms.

**Table 1:** Representative CRISPR/Cas-based strategies in starter cultures and industrial food microorganisms: technological outcomes, application relevance, and experimental validation context.

Microorganism/Strain	CRISPR/Cas strategy	Genetic target/modification	Main outcome	Application relevance/validation context	Reference
*Streptococcus thermophilus*	Endogenous CRISPR/Cas adaptive immunity	Acquisition of new spacers in the CRISPR1 locus following phage challenge.	Iterative spacer acquisition increased phage resistance; escape phages carried mutations or deletions in the targeted protospacers	Phage-resistance mechanism demonstrated in starter cultures; relevance to dairy culture selection	Deveau et al. (2008)
Industrial *Streptococcus thermophilus* starter cultures	Endogenous CRISPR/Cas-based phage immunization	Acquisition of 1–4 new CRISPR1 spacers following repeated phage challenge; an additional CRISPR3 spacer was acquired in one mutant.	Repeated phage challenge produced bacteriophage-insensitive derivatives with newly acquired spacers and improved culture stability	Phage immunization demonstrated in industrial dairy starter cultures; food-production performance not directly assessed	Mills et al. (2010)
*Streptococcus thermophilus* isolates from yoghurt	CRISPR profiling + phenotypic characterization	Comparative profiling of natural variation in CRISPR loci and spacer content; no genome editing was performed.	CRISPR profiles differentiated isolates that also varied in acidification, proteolytic, lipolytic and antimicrobial traits	Phenotypic characterization of yoghurt isolates; strain-selection evidence, not CRISPR genome editing	Coban et al. (2024)
*Streptococcus thermophilus* LMD-9	Repurposed endogenous type II-A CRISPR1/CRISPR3	Targeted deletion, nucleotide substitution and chromosomal insertion at selected loci, including genes of the eps cluster.	Precise edits were obtained at efficiencies of 75–100%; a 785-bp *lacZ* deletion reached 90% with CRISPR3 and a 1.5-kb repair template	Laboratory validation of an endogenous editing platform in a dairy starter; food-process performance not assessed	Ma et al. (2024)
*S. thermophilus* S-3	CRISPR/nCas9 seamless single- and multigene editing	Single- and multigene deletion of *epsA*, *epsB*, *epsC*, *epsE* and *epsG* in the EPS-biosynthesis pathway.	Selected deletions altered EPS molecular weight, viscosity and composition, enabling identification of key determinants of EPS formation and structure	Laboratory functional analysis of EPS biosynthesis and rheology-related traits; no food-matrix validation reported	Kong et al. (2022a)
*Saccharomyces cerevisiae*	CRISPR/Cas9 genome editing	Triple deletion of *ERG5*, *ERG4* and *ERG*3 to modify the sterol-biosynthesis pathway.	The triple mutant showed improved thermotolerance; ethanol production increased from 20.2 to 22.1 g/L at 37 °C and from 3.3 to 9.4 g/L at 42 °C	Fermentation performance evaluated under thermal stress; industrial relevance, without direct food-matrix validation	Yang et al. (2023)
*Saccharomyces cerevisiae*	Target-AID CRISPR base editing	Targeted diversification of the *SPT15* general-transcription-factor gene, generating 36 sequence variants.	Eighteen stress-tolerant mutants exhibited more than 1.5-fold higher fermentation capacity under the tested stress conditions	Laboratory selection of stress-tolerant mutants with improved fermentation capacity; food-matrix performance not reported	Liu Y. et al. (2021)
*S. cerevisiae* SPC-SNU 70-1 (commercial baker’s yeast)	Scarless CRISPR/Cas9 genome editing	Scarless editing of genes involved in glucose repression, glycerol biosynthesis and cellular redox balance.	The edited strains showed improved maltose utilization and leavening, enhanced osmotic- and freeze-stress tolerance, and better bread-storage properties	Direct food-process validation: improved leavening and stress tolerance under bread-making conditions	Cha et al. (2025)
*Saccharomyces cerevisiae* N1	Cas9-based genome editing	Double deletion of the *RGT2* and SNF3 glucose-sensor genes (ΔRGT2ΔSNF3).	Higher CO₂ production increased bread-dough volume by 18% compared with the parental strain	Direct food-matrix validation: improved CO₂ production and bread-dough volume	Lee et al. (2022)
*Lacticaseibacillus paracasei*	Endogenous CRISPR/Cas9 functional analysis	Targeted disruption of *glpF* and *glp*O, involved in glycerol metabolism and oxidative-stress response, for functional validation.	The functional roles of *glpF* and *glpO* in oxidative-stress tolerance under fermentation conditions were confirmed	Laboratory functional validation of oxidative-stress genes under fermentation-related conditions; improved industrial performance not demonstrated	Wu et al. (2025)

EPS, exopolysaccharides; LAB, lactic acid bacteria. Validation context distinguishes direct testing in food matrices or processing conditions from laboratory functional studies and strain-selection evidence.

### 3.2 CRISPR/Cas-based metabolic engineering of food-associated microorganisms

Metabolic engineering aims to redirect microbial metabolic flux toward desirable compounds while limiting the formation of competing or unwanted products. CRISPR/Cas technologies have broadened this approach by enabling precise modification of structural genes, regulatory elements, and competing pathways through targeted gene deletion, integration, replacement, or transcriptional regulation (Volk et al., 2023). This precision is particularly relevant to food biotechnology, where microbial metabolism determines not only fermentation efficiency but also the nutritional, sensory, functional, and safety properties of fermented foods (Hilgendorf et al., 2024; Auchtung et al., 2025). CRISPR-based metabolic engineering therefore offers a way to move beyond empirical strain selection toward the rational design of microorganisms with defined metabolic and technological functions (Choi and Lee, 2023; Yang et al., 2025).

#### 3.2.1 Engineering of fermentation and technologically relevant metabolites

One of the most direct applications is enhancing metabolite production that influences fermentation performance. Using the endogenous subtype II-A CRISPR/Cas system of *Pediococcus acidilactici* LA412, Liu L. et al. (2021) integrated an additional gene encoding L-lactate dehydrogenase into the chromosome to enhance lactic acid biosynthesis. The engineered strain showed increased lactic acid production and improved growth, demonstrating the potential of targeted pathway engineering to enhance fermentation-relevant traits in LAB. More extensive redistribution of fermentation metabolism was demonstrated in *Clostridium tyrobutyricum*, where Zhang et al. (2018) used the endogenous type I-B CRISPR/Cas system for single and multiplex genome editing. Replacing *cat1* with *adhE1*/*adhE2* redirected carbon flux toward butanol production, with editing efficiencies reaching 100%. These studies show how CRISPR/Cas systems can modify central metabolic branch points or add catalytic capacity to shift fermentation product distribution.

CRISPR-based engineering can also target metabolites that determine the technological properties of fermented foods. Exopolysaccharides (EPS) produced by lactic acid bacteria (LAB) are particularly relevant because they influence viscosity, texture, water retention, and mouthfeel and may reduce the need for externally added texturising agents. In *S. thermophilus* LMD-9, the endogenous type II-A CRISPR1 and CRISPR3 systems were repurposed to investigate the genetic basis of EPS biosynthesis. Targeted disruption of genes within the *eps* cluster identified *epsA*, *epsE*, and *epsG* as key determinants of EPS formation (Ma et al., 2024). Building on this pathway-level knowledge, Kong et al. (2022b) developed a CRISPR/dCas9-based regulatory system to optimize EPS biosynthesis in *S. thermophilus*. The system achieved 66–98% repression of individual or multiple target genes, while the combined repression of *galK* and overexpression of *epsA* and *epsE* increased EPS production approximately two-fold, reaching 277 mg/L. This study demonstrates how CRISPR-based regulation can coordinate precursor availability and pathway activity to enhance EPS production.

#### 3.2.2 Engineering of functional and bioactive metabolites

Another important application of CRISPR/Cas is the targeted modification of biosynthetic pathways to enhance the production of metabolites with nutritional or functional value. In *Lactiplantibacillus plantarum* WCFS1, Zhou et al. (2019) used CRISPR/Cas9-assisted recombineering with double- and single-stranded DNA to redirect metabolism toward N-acetylglucosamine (GlcNAc) production. Truncating nagB limited the competing conversion of glucosamine-6-phosphate, while a riboswitch replacement and a point mutation in *glmS1* alleviated regulatory constraints on pathway activity. By simultaneously reducing a competing reaction and relieving feedback regulation, the engineered strain produced 797.3 mg/L of GlcNAc without retaining exogenous genes or plasmids. This example demonstrates how CRISPR-assisted engineering can address multiple metabolic bottlenecks within the same pathway and generate strains with a comparatively limited genetic footprint.

CRISPR-based strategies have also enhanced gamma-aminobutyric acid (GABA) production, a bioactive metabolite relevant to functional foods. In *Levilactobacillus brevis* EG040, *gadR* overexpression increased GABA secretion by 37.9% and survival in simulated gastric fluid by 19%, whereas *norG* deletion reduced GABA secretion by 97.3% and impaired growth (Jo et al., 2026). In *S. cerevisiae*, deletion of the GABA-catabolic gene *UGA1* increased production by 104%, and fermentation optimisation yielded 280.36 mg/L (Ding et al., 2026). CRISPR/Cas9–RecT recombineering enabled the combinatorial modification of three metabolic genes in *Corynebacterium glutamicum* (Cho et al., 2017), whereas CRISPRi-based screening and host engineering, combined with food-grade expression of *gadA*, were used to enhance glutamate decarboxylase activity for GABA production in *Bacillus subtilis* (Zhang K. et al., 2025).

Vitamins are another important target for CRISPR-based metabolic engineering of food-associated microorganisms. Son et al. (2020) developed a CRISPR interference (CRISPRi) platform in *Leuconostoc citreum* and applied it to redirect metabolic flux toward riboflavin (vitamin B2) production. Repression of *folE*, which diverts the common GTP precursor toward folate biosynthesis, and *ribF*, which converts riboflavin to flavin mononucleotide, reduced their transcript levels by 5.6- and 3.3-fold, respectively. Simultaneous repression of both genes increased riboflavin production from 1.08 mg/L in the wild-type strain to 1.30 mg/L. Combining CRISPRi-mediated pathway regulation with rib operon expression increased riboflavin production to 1.7 mg/L, a 1.53-fold increase over the wild type.

Beyond nutritional and functional metabolites, CRISPR-based metabolic engineering may also enhance microbial functions relevant to food biopreservation. Anand et al. (2026) used CRISPR/Cas9 to disrupt *plnD*, a negative regulator of the plantaricin quorum-sensing network in *L. plantarum* 8P-A3. Loss of *plnD* increased expression of *plnA*, *plnE*, and *plnF* and was accompanied by enhanced antimicrobial activity. Although increased expression of plantaricin-associated genes does not by itself quantify bacteriocin production, the observed antimicrobial phenotype shows that CRISPR-mediated modification of regulatory circuits can enhance protective functions in food-associated bacteria.

#### 3.2.3 Reduction of undesirable fermentation metabolites

CRISPR-based metabolic engineering can also reduce undesirable compounds produced during food fermentation by modifying pathways responsible for their formation, accumulation, or degradation. Ethyl carbamate is a relevant example, as it can form in alcoholic beverages through reactions involving urea derived from yeast arginine metabolism. Chin et al. (2016) used CRISPR/Cas9 to introduce a nonsense mutation or delete *CAR1*, which encodes arginase, in *S. cerevisiae*. The engineered strains showed approximately 98% lower arginase activity, with 64.3–68.7% less urea formation and a 60.5–67.3% reduction in ethyl carbamate, without compromising ethanol fermentation performance. An alternative strategy was applied during Chinese rice wine fermentation. Wu et al. (2020) used an improved CRISPR/Cas9 system to integrate a DUR3 overexpression cassette at the *HO* locus in a previously modified *S. cerevisiae* strain. Increased *DUR3* expression enhanced urea utilization and reduced urea and ethyl carbamate concentrations by 92.0 and 58.5%, respectively, compared with the original N85 strain, with negligible effects on fermentation performance. Jung et al. (2023) subsequently deleted CAR1 and *GZF3* in a polyploid *S. cerevisiae* starter, reducing ethyl carbamate formation by 41.6% during Makgeolli fermentation without significantly affecting ethanol production or the overall fermentation profile. These studies show that CRISPR-based pathway engineering can improve food safety by reducing undesirable microbial metabolic products.

### 3.3 CRISPR/Cas engineering of probiotic and beneficial microorganisms

Probiotic microorganisms are an emerging target for CRISPR/Cas-based engineering (Binda et al., 2020; Zuo and Marcotte, 2021; Liu et al., 2023). In this context, CRISPR technologies can facilitate functional characterization of genetic determinants underlying probiotic traits and enable the rational construction of strains with defined or enhanced functions (Zuo and Marcotte, 2021). Traits such as survival under gastrointestinal conditions, adhesion and persistence, metabolic activity, and interactions with the host or resident microbiota are highly strain-dependent and often genetically complex (Liu et al., 2023). Precise genome editing therefore provides an important tool for establishing genotype–phenotype relationships that may subsequently guide the development of improved probiotic strains (Pan and Barrangou, 2020; Xie et al., 2026).

#### 3.3.1 Development of CRISPR/Cas editing platforms in probiotic microorganisms

Initial studies have largely focused on developing efficient genome-editing platforms in probiotic microorganisms that have traditionally been difficult to manipulate genetically (Zuo and Marcotte, 2021; Liu et al., 2023). Hidalgo-Cantabrana et al. (2019) repurposed the endogenous type I-E CRISPR-Cas system of *Lactobacillus crispatus* for targeted genome editing. The system enabled a range of modifications, including a 643-bp deletion in the gene encoding an exopolysaccharide priming glycosyltransferase, achieved with 100% efficiency, as well as a stop-codon insertion, a single-nucleotide substitution, deletion of a prophage-associated region, and chromosomal integration of *gfp*. Although this study primarily established an editing platform rather than an improved probiotic phenotype, it demonstrated that endogenous CRISPR systems can overcome genetic limitations in previously recalcitrant beneficial microorganisms and provide a basis for subsequent functional engineering.

The range of genome-engineering strategies available for probiotic and beneficial bacteria has since expanded to include Cas9 nickases, Cas12a, CRISPR interference (CRISPRi), base editors, ribonucleoprotein (RNP)-mediated editing, prime editing, and repurposed endogenous systems (Liu et al., 2023; Wiull et al., 2025; Mitsunobu et al., 2025; Chen et al., 2025; Kim et al., 2025). For example, CRISPR/Cas9-assisted recombineering enabled highly efficient genome modification in *Limosilactobacillus reuteri* (Oh and van Pijkeren, 2014), whereas repurposing an endogenous type II-A CRISPR-Cas9 system subsequently enabled a 40-kb deletion and chromosomal integration of a 1743-bp sequence in this species (Guo et al., 2023). In *Clostridium butyricum*, an endogenous type I-B system enabled seamless gene deletion with efficiencies of up to 100% (Zhou et al., 2021), while subsequent Cas12a-based platforms enabled targeted genome modifications and plasmid curing (Zhang et al., 2023; Li D. et al., 2026). Li D. et al. (2026) further integrated CRISPR-Cas12a editing with an anaerobic fluorescent reporter system, thereby expanding the toolkit for metabolic engineering under strictly anaerobic conditions. Together, these advances mark a transition from proof-of-concept genome editing to versatile CRISPR platforms tailored to diverse probiotic species and strains (Zuo and Marcotte, 2021; Liu et al., 2023).

An important recent development in microbial genome engineering is the emergence of base and prime editing as alternatives to conventional double-strand-break (DSB)-dependent CRISPR/Cas approaches (Liu et al., 2023; Mitsunobu et al., 2025; Chen et al., 2025). Base editors enable targeted nucleotide conversions without introducing DSBs, whereas prime editors allow defined nucleotide substitutions and small insertions or deletions (Mitsunobu et al., 2025; Chen et al., 2025). Recent studies have demonstrated the feasibility of these approaches in beneficial bacterial hosts. For example, CRISPR-based base-editing systems have been developed for lactobacilli and bifidobacteria (Mitsunobu et al., 2025; Lin et al., 2025), while prime editing has been demonstrated in the probiotic strain *E. coli* Nissle 1917 (Chen et al., 2025). These approaches may be particularly valuable for genetically recalcitrant probiotic bacteria, where limited homologous recombination and poor survival after DSB formation can constrain conventional Cas9-mediated editing (Liu et al., 2023; Chen et al., 2025). Nevertheless, these approaches present several technical challenges, including PAM dependency, restricted editing windows, unintended bystander substitutions (particularly in base editing), and potential off-target effects. Prime editing additionally requires careful prime-editing guide RNA (pegRNA) design, and its efficiency may depend on host-specific DNA repair mechanisms (Chen et al., 2025). Furthermore, inefficient delivery of editing components and strain-dependent transformation efficiency remain important obstacles to broader application in probiotic microorganisms (Liu et al., 2023; Lin et al., 2025). Consequently, the efficiency, specificity and reproducibility of precision genome-editing approaches require validation in individual probiotic strains.

#### 3.3.2 CRISPR/Cas-based functional genomics of probiotic traits

CRISPR/Cas has subsequently been applied to investigate specific traits associated with probiotic fitness. In *L. plantarum* AR113, targeted gene-knockout studies have been used to dissect the genetic determinants of bile resistance and intestinal colonization (Wang et al., 2021; Qin et al., 2022). Wang et al. (2021) used CRISPR/Cas9 to construct single-, double-, and triple-knockout mutants of genes encoding bile salt hydrolases. Functional analysis showed that *bsh1* and *bsh3* mainly contribute to BSH activity and bile salt resistance in this strain, especially during exposure to glycine-conjugated bile salts. In a separate study, Qin et al. (2022) used CRISPR/Cas9 to delete putative mucin- and adhesion-associated genes in the same strain, establishing a relationship between bacterial adhesion and intestinal persistence. Combined deletion of *Lp_1431*, *Lp_2233*, and *Lp_2792* reduced adhesion to HT-29 cells from 175 to 114 bacteria per 100 cells and shortened intestinal colonization in mice from 14 to 11 days, while having little effect on acid and bile tolerance. These findings are particularly important because they demonstrate CRISPR as a functional genomics tool for identifying determinants of probiotic colonization, rather than directly showing CRISPR-mediated enhancement of colonization. Together, these knockout studies show how CRISPR-based functional genomics can identify targets for improving probiotic fitness.

In contrast, CRISPR has been used directly in probiotic strains relevant to functional foods. Xie et al. (2026) exploited the endogenous type II-A CRISPR-Cas system of the widely used probiotic *Lacticaseibacillus rhamnosus* GG (LGG), enabling targeted deletions and chromosomal insertions with editing efficiencies of 11.1–25.0% among recovered transformants. In this study, a *gusA* expression cassette was integrated into the chromosome to generate a stable β-glucuronidase-positive LGG strain that could be tracked in complex microbial communities and animal models. Although the introduced reporter did not enhance probiotic efficacy, the platform enables investigation of persistence, fitness, and host–microbiota interactions and establishes a foundation for future construction of LGG strains with defined functional properties. Substantial progress has also been achieved in *Bifidobacterium*. More than 95% of analyzed *Bifidobacterium animalis* subsp. *lactis* genomes harbor a conserved type I-G CRISPR-Cas system, which Raftopoulou et al. (2026) adapted across six commercial strains. Deletion of glycoside-hydrolase genes in the Balac_1593-Balac_1601 cluster showed that the GH36 alpha-galactosidase encoded by *Balac_1601* is required for melibiose and raffinose utilization. CRISPRi has additionally enabled transcriptional analysis of nucleotide, carbohydrate, and exopolysaccharide metabolism across several *Bifidobacterium* species (Friess et al., 2025), while cytosine base editors support double-strand-break-free modification in multiple probiotic taxa (Pan et al., 2022; Lin et al., 2025). These platforms help overcome long-standing constraints associated with transformation efficiency, cell-envelope properties, and strain-specific restriction-modification systems (Shin and Barrangou, 2026).

#### 3.3.3 Engineering protective and bioactive functions

Although some of the following examples concern biomedical rather than food applications, they illustrate the broader technological potential of CRISPR/Cas engineering in probiotics and other beneficial microorganisms. CRISPR systems can also confer protective functions on probiotic microorganisms. Fang et al. (2024) integrated a type I-E CRISPR-Cas3 system from *E. coli* BW25113 into *E. coli* Nissle 1917 (EcN), which lacks a complete and functional endogenous CRISPR-Cas system. Programmed targeting of *mcr-1*, *blaNDM-1*, and *tet(X)* limited the acquisition of these antimicrobial-resistance determinants *in vitro* and in the zebrafish intestine, demonstrating CRISPR-Cas as an engineered defense rather than a conventional editing tool. These findings provide proof of concept for a protective function in experimental models, but do not demonstrate effectiveness in food-related applications. Chen et al. (2025) subsequently adapted prime editing to EcN by combining a Cas9 nickase, a reverse transcriptase, and a prime-editing guide RNA. The system enabled substitutions, insertions, and deletions without conventional double-strand breaks and was used for genomic barcoding and to establish an antibiotic-resistance-gene-free platform. These results demonstrate the feasibility of precise and traceable genome engineering in a probiotic strain, although improved probiotic efficacy or performance in food systems was not established.

A further step toward designer probiotics is engineering strains to deliver defined bioactive functions. Liu et al. (2026) characterized a compact type II-C Cas9 (BLCas9) from *Bifidobacterium longum* subsp. longum and used it to establish a dual-plasmid genome-editing platform in EcN. This system enabled targeted metabolic engineering that enhanced extracellular GABA production, demonstrating the feasibility of introducing a defined metabolic function into a probiotic microorganism. However, increased metabolite production under experimental conditions does not, by itself, establish functional performance in a food matrix or a health benefit following consumption. Tang et al. (2026) reported a related approach for *Lactococcus lactis* NZ9000, combining CRISPR/Cas9-mediated chromosomal knock-in with Cre/loxP-based genome reduction to construct a strain that stably expresses a high-activity uricase variant. The engineered strain completely degraded approximately 500 μM urate within 20 h and persisted moderately in the murine gastrointestinal tract. These findings support its potential as a living biotherapeutic candidate, although its technological suitability and performance in food production have not been evaluated. More broadly, these studies illustrate the potential of CRISPR/Cas to introduce protective and bioactive functions into beneficial microorganisms, while underscoring the distinction between findings from laboratory or biomedical models and applications validated in food biotechnology.

#### 3.3.4 Safety-oriented engineering of probiotic strains

CRISPR-based engineering may also improve probiotic safety by eliminating undesirable genetic traits. Pan et al. (2022) repurposed the endogenous type I-G CRISPR-Cas system and a cytosine base editor in *B. animalis* subsp. *lactis*. Targeted deletion of *tetW* and base-editing strategies restored tetracycline susceptibility in several strains, demonstrating the potential of CRISPR-based approaches to remove undesirable antimicrobial resistance phenotypes from probiotic bacteria. Safety-oriented engineering has also been explored using transient Cas9–gRNA RNP complexes in *L. plantarum* and *L. brevis*. Kim et al. (2025) introduced a 50-bp deletion and a premature stop codon into *hlyIII* in *L. plantarum* WCFS1, *L. plantarum* SPC 72-1, and *L. brevis* SPC-SNU 70-2. Compared with the corresponding wild-type strains, the resulting mutants showed reductions in free hemoglobin levels of 27.0%, 74.3%, and 5.0%, respectively. These strain-dependent effects suggest that *hlyIII* inactivation may contribute to reduced hemolytic activity. However, because *hlyIII* has not yet been conclusively shown to encode a functional hemolysin in these species, its specific contribution to the observed phenotype remains to be confirmed (Kim et al., 2025). These studies broaden the scope of CRISPR-based probiotic engineering from enhancing desirable traits to identifying and potentially eliminating genetic determinants of undesirable phenotypes.

Table 2 summarizes representative CRISPR/Cas-based engineering strategies in probiotic and beneficial microorganisms and their functional outcomes.

**Table 2:** Representative CRISPR/Cas-based engineering strategies in probiotic and beneficial microorganisms: functional outcomes, application relevance, and experimental validation context.

Microorganism/Strain	CRISPR/Cas strategy	Genetic target/modification	Main outcome	Relevance to probiotic/validation context	Ref.
*Lactobacillus crispatus* NCK1350	Endogenous type I-E CRISPR-Cas	Targeted deletions, nucleotide substitution and chromosomal insertion, including p-*gtf* editing	643-bp p-*gtf* deletion reached 100% efficiency; stop-codon insertion, single-nucleotide substitution, prophage-region deletion and *gfp* integration were also achieved	Established an endogenous editing platform for functional genomics and engineering of a beneficial/probiotic-associated species/without demonstrated food-related performance	Hidalgo-Cantabrana et al. (2019)
*Limosilactobacillus reuteri* ATCC PTA 6475	CRISPR-Cas9-assisted ssDNA recombineering	Sequence-specific chromosomal edits at multiple loci	CRISPR counterselection increased recovery of desired edits to 90–100% for three tested targets and enabled codon-level mutagenesis	High-efficiency genome-engineering platform for a probiotic LAB/ no food-matrix assessment	Oh and van Pijkeren (2014)
*Limosilactobacillus reuteri* 03	Endogenous type II-A CRISPR-Cas9	Gene deletion, point mutation, large-fragment deletion and gene integration	Editing efficiencies reached 95.46% for deletion and 86.36% for point mutation; a 40-kb deletion and 1,743-bp integration (73.9%) were demonstrated	Advanced endogenous editing platform for development of a probiotic chassis and live biotherapeutic products/ without demonstrated functional efficacy	Guo et al. (2023)
*Lacticaseibacillus casei* LC2W	CRISPR-Cas9D10A nickase (pLCNICK)	In-frame deletion of four genes and chromosomal eGFP insertion	Targeted deletions and insertions were achieved with efficiencies of 25–62% in a one-step workflow	Rapid and precise genome engineering in a health-promoting and food-associated LAB/ no technological phenotype or food-matrix validation	Song et al. (2017)
*Lactiplantibacillus plantarum* AR113	CRISPR-Cas9	Single and multiple knoc-kout of *bsh* genes	*bsh* genes differentially affected growth in bile-containing media, aggregation, surface properties and adhesion to HT-29 cells; *bsh2* was linked to adhesion	Functional identification of genetic determinants relevant to gastrointestinal fitness and colonization/ no evidence of improved probiotic efficacy	Wu et al. (2022)
*Lactiplantibacillus plantarum* AR113	CRISPR-Cas9	Deletion of mucin/adhesion-associated genes, including Lp_1431, Lp_2233 and Lp_2792; chromosomal RFP labeling	Triple deletion reduced adhesion to HT-29 cells from 175 to 114 bacteria/100 cells and shortened intestinal persistence in mice from 14 to 11 days; acid and bile tolerance were largely unchanged	Functional dissection of determinants of adhesion and intestinal persistence/ not food-process validation	Qin et al. (2022)
*Lactiplantibacillus plantarum* WCFS1 and SPC 72-1; *Levilactobacillus brevis* SPC-SNU 70–2	Cas9-gRNA ribonucleoprotein (RNP) + RecE/T	50-bp deletion and stop-codon introduction in *hlyIII*	*ΔhlyIII* mutants were obtained in all three strains; free hemoglobin levels decreased by 27.0%, 74.3 and 5.0%, respectively, although the functional role of *hlyIII* requires further validation	Demonstrates transient RNP-based Cas9 delivery that reduces reliance on plasmid-borne CRISPR components; the functional role of *hlyIII* and its relevance to strain safety require further validation	Kim et al. (2025)
*Lactiplantibacillus plantarum* WCFS1	Inducible two-plasmid CRISPR-Cas9 knock-in system	Chromosomal insertion of *mCherry* reporter and SARS-CoV-2 receptor-binding domain expression cassettes (~0.8–1.3 kb)	Stable chromosomal insertion enabled inducible or constitutive expression of heterologous proteins in the cytoplasm or on the cell surface	Demonstrates the capacity of *L. plantarum* to serve as a genetically stable delivery chassis for functional heterologous proteins/ not food-related validation	Wiull et al. (2025)
*Clostridium butyricum*	Heterologous type II CRISPR-Cas9 and endogenous type I-B CRISPR-Cas	Targeted deletion of *spo0A* and *aldh*	The endogenous type I-B system enabled seamless deletion with efficiencies up to 100%; heterologous Cas9 imposed a strong transformation burden	Expanded the genetic toolbox for a probiotic anaerobe and highlighted advantages of endogenous systems/ technological performance not assessed	Zhou et al. (2021)
*Clostridium butyricum*	CRISPR-Cas12a	Scarless genome manipulation, anaerobic reporter system, plasmid curing and acetaldehyde-pathway engineering	Markerless editing was established; *aldh* overexpression increased enzyme activity by 79.29% as a proof of concept	Advanced platform for functional and metabolic engineering under strict anaerobic conditions/ no food-matrix validation	Li D. et al. (2026)
*Bifidobacterium animalis* subsp. *lactis*	Endogenous type I-G CRISPR-Cas and CRISPR cytosine base editor	500-bp deletion and nonsense mutation of *tetW*; additional genomic edits	*tetW* deletion abolished tetracycline resistance; C-to-T base editing restored tetracycline susceptibility in multiple strains	Direct example of removing an undesirable antimicrobial-resistance phenotype from probiotic bifidobacteria/ food-use safety requires separate evaluation	Pan et al. (2022)
*Bifidobacterium animalis* AR668	CRISPR-Cas9	Knockout of genes 0348 and 0208; homology-arm optimisation and inducible plasmid curing	Successful targeted knockouts and establishment of an inducible plasmid-curing system	Provides a platform for functional-genetic analysis and rational modification of bifidobacteria/without food-process validation	Li et al. (2023)
*Bifidobacterium longum*, *B. adolescentis*, *B. pseudocatenulatum* and related strains	CRISPR-Cas9 cytosine base editor (cBEST)	DSB-free C-to-T base editing; disruption of conserved metabolic targets	Restriction-modification bypass markedly improved editing efficiency, and the same editing design was portable across several *Bifidobacterium* species	Expands precision editing for probiotic bifidobacteria while avoiding double-strand breaks/ no direct food-related functional performance demonstrated	Lin et al. (2025)
*Bifidobacterium breve* and other bifidobacterial species	CRISPRi using nuclease-dead Cas9 orthologs	Targeted repression of reporter genes and endogenous genes involved in nucleotide and carbohydrate metabolism	A one-plasmid CRISPRi platform achieved efficient gene repression across multiple bifidobacterial species without extensive strain-specific optimisation	Portable functional-genomics platform for commercially important and health-associated bifidobacteria/functional-genomics tool rather than a food-application study	Friess et al. (2025)
*Bifidobacterium animalis* subsp. *lactis* (six commercial strains)	Endogenous type I-G CRISPR-Cas	Knockout of glycoside hydrolases within the *Balac_1593–Balac_1601* cluster	Editing was validated across six commercial strains; *Balac_1601* deletion abolished melibiose and raffinose utilization, and sequential editing was demonstrated	Enables genotype–phenotype mapping and future strain improvement directly in commercial probiotic strains; no food-matrix performance assessment	Raftopoulou et al. (2026)
*Lacticaseibacillus rhamnosus* GG (LGG)	Endogenous type II-A CRISPR-Cas	Targeted deletions and insertions; chromosomal *gusA* integration	Editing efficiencies of 11.1–25.0% were obtained, and a stable GUS-positive LGG reporter strain was generated for tracking in complex communities	Enables investigation of probiotic persistence and provides a platform for functional strain construction/ no demonstrated improvement in probiotic efficacy	Xie et al. (2026)
*Levilactobacillus brevis* EG040	CRISPR/Cas9 genome editing	*gadR* overexpression; *norG* deletion	*gadR* overexpression increased GABA secretion by 37.9% and survival in simulated gastric solution by 19%; *norG* deletion reduced GABA secretion by 97.3% and impaired growth	Engineering of a food-derived GABA-producing LAB; links GABA biosynthesis with a probiotic-relevant stress-resistance phenotype/no direct validation in a food matrix reported	Jo et al. (2026)
*Escherichia coli* Nissle 1917 (EcN)	Engineered type I-E CRISPR-Cas3	CRISPR targeting of *mcr-1*, *blaNDM-1* and tet(X)	Engineered EcN efficiently blocked acquisition of targeted ARGs *in vitro* and showed immunity to their transfer in the zebrafish intestine	CRISPR itself provides a defined protective function to a probiotic chassis/ no food-system validation	Fang et al. (2024)
*Escherichia coli* Nissle 1917 (EcN)	Prime editing (Cas9 nickase + reverse transcriptase + pegRNA)	Precise substitutions, insertions and deletions; genomic barcoding; ARG-free chassis construction	Prime editing enabled precise genome modification and construction of a barcoded, antibiotic-resistance-gene-free probiotic platform	Extends probiotic engineering to programmable precision editing with a limited genomic footprint/probiotic efficacy and food-related performance not evaluated	Chen et al. (2025)
*Escherichia coli* Nissle 1917 (EcN)	Compact BLCas9 from a type II-C system of *Bifidobacterium longum*	Dual-plasmid genome editing and targeted metabolic engineering	Robust/multiplex editing was established, and extracellular GABA production was enhanced	Designer-probiotic example combining a probiotic chassis with an engineered bioactive metabolic function/food-matrix performance and health effects not established	Liu et al. (2026)
*Lactococcus lactis* NZ9000	Inducible CRISPRi (dCas9)	Single/multiple gene repression; identification of LLNZ_07335 as a bile salt hydrolase	CRISPRi repressed gene expression by up to 99%; it identified a functional determinant of bile salt resistance	Links functional genomics with a probiotic-relevant stress-tolerance trait in an important food-grade LAB/no food-process validation	Xiong et al. (2020)
*Lactococcus lactis* NZ9000	Chromosomal and plasmid-based inducible CRISPRi	Conditional silencing of *acmA* and the essential gene *ybeY*	Chromosomal dCas9 integration reduced expression ~20-fold relative to plasmid expression, minimized leakage and enabled tightly controlled silencing of essential genes	Tunable functional-genomics platform for food-grade and beneficial *L. lactis*/not demonstrated food-related performance	Huang et al. (2025)
*Lactococcus lactis* NZ9000	CRISPR-Cas9 knock-in +*Cr*e/*loxP* genome reduction	Chromosomal integration of a high-activity uricase variant and genome streamlining	The engineered strain reached 2.34 U/mL uricase activity and completely degraded ~500 μM urate within 20 h; moderate colonization was observed in mice	Functional food-grade probiotic chassis with a defined therapeutic metabolic activity; relevant to designer probiotics/live biotherapeutics/ not a validated food application	Tang et al. (2026)

The validation context describes the experimental setting reported for each study; using a food-associated microorganism does not, by itself, demonstrate performance in food production or food matrices. ARG, antimicrobial-resistance gene; Cas, CRISPR-associated protein; CRISPR, clustered regularly interspaced short palindromic repeats; dCas9, nuclease-deficient Cas9; DSB, double-strand break; EcN, *Escherichia coli* Nissle 1917; GABA, γ-aminobutyric acid; GH, glycoside hydrolase; GUS, β-glucuronidase; LGG, *Lacticaseibacillus rhamnosus* GG; pegRNA, prime-editing guide RNA; RBD, receptor-binding domain; RNP, ribonucleoprotein.

### 3.4 CRISPR/Cas-based antimicrobial strategies against foodborne pathogens

Foodborne pathogenic bacteria remain a major challenge throughout food production and processing, and the rising prevalence of antimicrobial resistance (AMR) further complicates their control. Conventional antimicrobials typically target conserved cellular processes and may therefore affect both target and non-target microorganisms (Bikard et al., 2014; Citorik et al., 2014). CRISPR/Cas systems offer a fundamentally different strategy because sequence-specific recognition can program their antimicrobial spectrum (Gomaa et al., 2014). Depending on the selected Cas effector, genetic target, and delivery platform, CRISPR-based approaches can selectively eliminate bacterial cells, remove or inactivate resistance determinants, attenuate virulence, or facilitate the construction of engineered bacteriophages with enhanced antimicrobial properties (Yosef et al., 2015). These characteristics have stimulated interest in CRISPR/Cas as a potential precision tool for controlling foodborne pathogens, although most applications remain at the proof-of-concept or preclinical stage (Stout et al., 2017; Khosrojerdi et al., 2026).

Early studies established the feasibility of sequence-specific CRISPR antimicrobials using Cas9. Citorik et al. (2014) showed that RNA-guided nucleases delivered by phagemid particles or a conjugative plasmid could selectively eliminate *E. coli* carrying defined resistance or virulence genes. Similarly, Bikard et al. (2014) developed a phagemid-delivered Cas9 antimicrobial against *Staphylococcus aureus*. Chromosomal targeting caused sequence-specific killing, whereas targeting a plasmid-borne antibiotic-resistance gene eliminated the plasmid and prevented subsequent acquisition of the corresponding resistance plasmid. The system selectively depleted targeted bacteria from mixed populations and reduced their abundance in a mouse skin-colonization model. Together, these studies demonstrated that CRISPR antimicrobials can selectively remove pathogenic or resistant bacterial subpopulations while sparing genetically non-targeted cells. Kiga et al. (2020) later extended this concept by packaging CRISPR/Cas13a and programmable crRNAs into bacteriophage-derived capsids. The resulting CapsidCas13a antimicrobials selectively killed carbapenem-resistant *E. coli* and *mecA*-positive methicillin-resistant *S. aureus* (MRSA). Because Cas13a targets RNA, it can be bactericidal regardless of whether the recognized resistance gene is on the chromosome or a plasmid, provided the target transcript is expressed. By contrast, Cas9-mediated cleavage of a non-essential resistance plasmid may eliminate the plasmid without necessarily killing the bacterial cell. These findings show that the selected Cas effector and the target’s genomic location can substantially influence the antimicrobial outcome.

The antimicrobial performance of CRISPR systems depends on nuclease selection, guide design, and bacterial genotype. Vialetto et al. (2024) compared six CRISPR nucleases in multidrug-resistant and hypervirulent *Klebsiella pneumoniae* and found that DNA-targeting nucleases generally outperformed Cas13a, with AsCas12a showing robust activity across strains. However, guide efficiency and escape mechanisms varied by target and strain, indicating that sequence complementarity alone does not ensure effective bacterial elimination. CRISPR systems can also eliminate AMR determinants without necessarily killing the bacterial host. Nittayasut et al. (2024) used non-replicative phagemid particles to deliver CRISPR/Cas9 targeting major blaCTX-M variants in *E. coli*. The system promoted plasmid clearance and reduced the proportion of resistant cells by approximately 3–4 log₁₀. These findings support CRISPR’s potential to control transferable resistance determinants, although its effectiveness in food matrices remains to be established.

An emerging extension of precision antimicrobial engineering involves reducing bacterial virulence without eliminating the targeted population. Ronda et al. (2025) developed BACTRINS, a conjugatively delivered CRISPR-associated transposase platform that disrupted Shiga toxin genes while introducing a therapeutic nanobody payload, reducing pathogen virulence and improving survival in a murine infection model. Although BACTRINS was developed for *in situ* gut microbiome engineering rather than food decontamination, it provides mechanistic and preclinical evidence for programmable attenuation of bacterial virulence, without establishing efficacy in food matrices or food-processing environments (Ronda et al., 2025).

CRISPR interference offers a complementary approach for identifying and modulating virulence-associated functions. In the major foodborne pathogen *Campylobacter jejuni*, Costigan et al. (2022) established a dCas9-based CRISPRi system and demonstrated targeted repression of endogenous genes, including *astA* and the flagellar regulator *flgR*. Partial repression of *flgR* was achieved in two *C. jejuni* strains, enabling investigation of genes associated with motility and other phenotypes relevant to colonization and pathogenicity. In this context, CRISPRi should be regarded primarily as a functional-genomics platform rather than a deployable antimicrobial treatment. Nevertheless, identifying genetic determinants that can be transcriptionally suppressed without extensive genome modification may yield targets for future pathogen-attenuation strategies.

A particularly promising direction is to use CRISPR/Cas not as an antimicrobial cargo but as a tool to engineer bacteriophages with additional antimicrobial functions. Hupfeld et al. (2018) used the LivCRISPR-1 type II-A system from *Listeria ivanovii* to establish a genome-engineering platform for large, non-integrating *Listeria* phages. Using CRISPR/Cas-mediated counterselection, they engineered the broad-host-range phage A511 to express lysostaphin, a cell-wall hydrolase that targets *Staphylococcus* peptidoglycan. In bacterial co-culture, the engineered phage killed its *Listeria* host, and lysostaphin released after host-cell lysis also killed *S. aureus*. This laboratory co-culture study demonstrates that CRISPR-assisted phage engineering can extend antimicrobial activity beyond the phage’s natural host range, providing a mechanistic basis for the potential control of food-associated pathogens without demonstrating efficacy in food systems.

CRISPR/Cas9 was also used to engineer *Salmonella*-targeting bacteriophages. Woudstra et al. (2023) deleted the portal vertex or major capsid genes from the virulent phage S117 and several temperate *Salmonella* phages. These modifications prevented complete phage head formation and produced non-replicative tailocin particles that retained bactericidal activity against their native hosts. This study illustrates a distinct application of CRISPR/Cas9 as an engineering tool to convert naturally occurring phages into receptor-specific antimicrobial particles, rather than as an antimicrobial system delivered directly to bacterial cells. However, their effectiveness and stability in complex food matrices remain to be established.

### 3.5 CRISPR-based detection and biofilm control in food manufacturing

Microbial contamination during food production and processing remains a major challenge for food safety, product quality, and shelf life. Rapid identification of foodborne pathogens is essential to prevent contaminated products from entering the food chain, while persistent microbial populations in biofilms may contribute to recurrent contamination of food-contact surfaces and processing equipment. CRISPR/Cas technologies are being explored in both areas, although their technological maturity differs substantially. CRISPR-based diagnostics have advanced toward sensitive detection in complex food matrices and portable analytical formats (Shin et al., 2022; Azhar et al., 2026), whereas CRISPR-mediated biofilm control remains largely at the functional genomics and proof-of-concept stages (Elbehiry and Alajaji, 2026).

#### 3.5.1 CRISPR-based detection of foodborne pathogens

The diagnostic potential of CRISPR systems largely depends on the collateral nucleic-acid cleavage activity of Cas12 and Cas13 effectors following sequence-specific target recognition (Gootenberg et al., 2017; Chen et al., 2018). When combined with amplification methods such as recombinase polymerase amplification (RPA), recombinase-aided amplification (RAA), or other isothermal amplification strategies, this property enables highly sensitive detection without conventional thermal cycling. Importantly, recent studies have moved beyond purified nucleic acids to validate these methods in real-world food matrices. Lv et al. (2022) developed a one-pot CE–RAA–CRISPR/Cas12a assay for *Vibrio parahaemolyticus* in seafood. Chemical enhancement of the Cas12a reaction produced detection limits of 6.7 × 10^1^ CFU/mL in bacterial cultures and 7.3 × 10^1^ CFU/g in shrimp. The assay showed no cross-reactivity with other tested pathogens and achieved 100% diagnostic agreement with qPCR in food samples. These results show that CRISPR-based detection can retain analytical performance in a complex food matrix, not only under simplified laboratory conditions.

Cas13-based detection offers a complementary strategy for RNA recognition. Shi et al. (2025) developed the SHERLOCK-LM assay specifically for *Listeria monocytogenes* detection in food and evaluated it using 550 representative samples, including pre-enriched and non-enriched preparations. The optimized Cas13a-based assay detected as little as 1.5 fg of target and operated at 37–42 °C with reaction times of 5–40 min. Its performance was compared with conventional plate counting and RPA, and SHERLOCK-LM showed improved overall detection performance relative to RPA in complex food matrices. This study is particularly relevant because it shows progress from analytical proof of concept to broader validation using heterogeneous food samples and workflows that reduce dependence on prolonged enrichment. Further development increasingly focuses on simplifying sample processing and integrating CRISPR detection into portable platforms suitable for on-site testing. Zheng et al. (2025) developed a single-tube ST-EXPAR-CRISPR assay for *Salmonella enterica serovar Typhimurium/Enteritidis* that combined bacterial lysis, exponential amplification, and CRISPR/Cas12a detection in a single reaction at 39 °C. The system eliminated separate bacterial isolation, nucleic acid extraction, and washing steps, achieving a detection limit of 37 CFU/mL. Notably, untrained volunteers successfully used the kit and portable device to detect contamination in food samples outdoors, demonstrating that simplifying CRISPR workflows can reduce technical barriers to field deployment.

Microfluidic integration marks another important step toward practical food-safety monitoring (Xing et al., 2023). Han et al. (2026) combined RPA–CRISPR/Cas12a detection with a reusable, finger-actuated microfluidic device incorporating a modular pressurizing pump. The platform was configured to detect *E. coli* O157: H7, *Salmonella* spp., and *L. monocytogenes*, and was validated using pathogen-spiked milk, with detection limits of 1.62, 1.84, and 1.01 CFU/mL, respectively. The reconfigurable architecture enables flexible, multiplexed testing without conventional laboratory instrumentation. Tian et al. (2026) further developed a pump-free CRISPR/Cas12a microfluidic chip for simultaneous detection of *L. monocytogenes*, *S. aureus*, *E. coli* O157:H7, and *Cronobacter sakazakii*, with sensitivities of 10^2^-10^3^ CFU/mL, depending on the pathogen. Together, these studies illustrate the shift from single-target assays to integrated multiplexed surveillance.

Another important direction is developing CRISPR-based diagnostic methods that reduce or eliminate the need for target nucleic acid amplification. Zhang X. et al. (2025) developed a CRISPR/Cas12a-mediated magnetic relaxation switching biosensor for amplification-free detection of *Salmonella enterica serovar Typhimurium/Enteritidis*. Target recognition activated Cas12a trans-cleavage and an enzymatic signal-amplification cascade that generated a magnetic relaxation response. The assay covered a dynamic range of 40–10^7^ CFU/mL and achieved a detection limit of 10 CFU/mL without target nucleic-acid amplification. Validation in food samples showed strong agreement with qPCR (*R*^2^ = 0.989). This approach could simplify analytical workflows and reduce the risk of amplicon contamination, although broader validation across different food matrices remains necessary. CRISPR diagnostics are also beginning to extend beyond food-product analysis to monitoring food-processing environments. Jeong et al. (2026) combined an electrostatic air sampler with an RPA–CRISPR/Cas12a assay in a field-deployable platform to detect airborne *S. aureus*, *Salmonella enterica serovar Typhimurium/Enteritidis*, *L. monocytogenes*, and *Bacillus cereus*. The respective detection limits were 274.9, 4.5, 9.5, and 28.5 CFU/mL, with detection completed within 45 min and the overall analytical phase requiring approximately 1 h. This approach extends CRISPR-based surveillance beyond end-product testing and demonstrates its potential for on-site monitoring of airborne contamination in food-processing environments. Collectively, these advances illustrate the development of increasingly portable, integrated, multiplexed, and amplification-free CRISPR diagnostic platforms.

Figure 3 summarizes major CRISPR-based strategies for pathogen detection and biofilm control in food manufacturing, along with their different levels of technological maturity.

**Figure 3:**
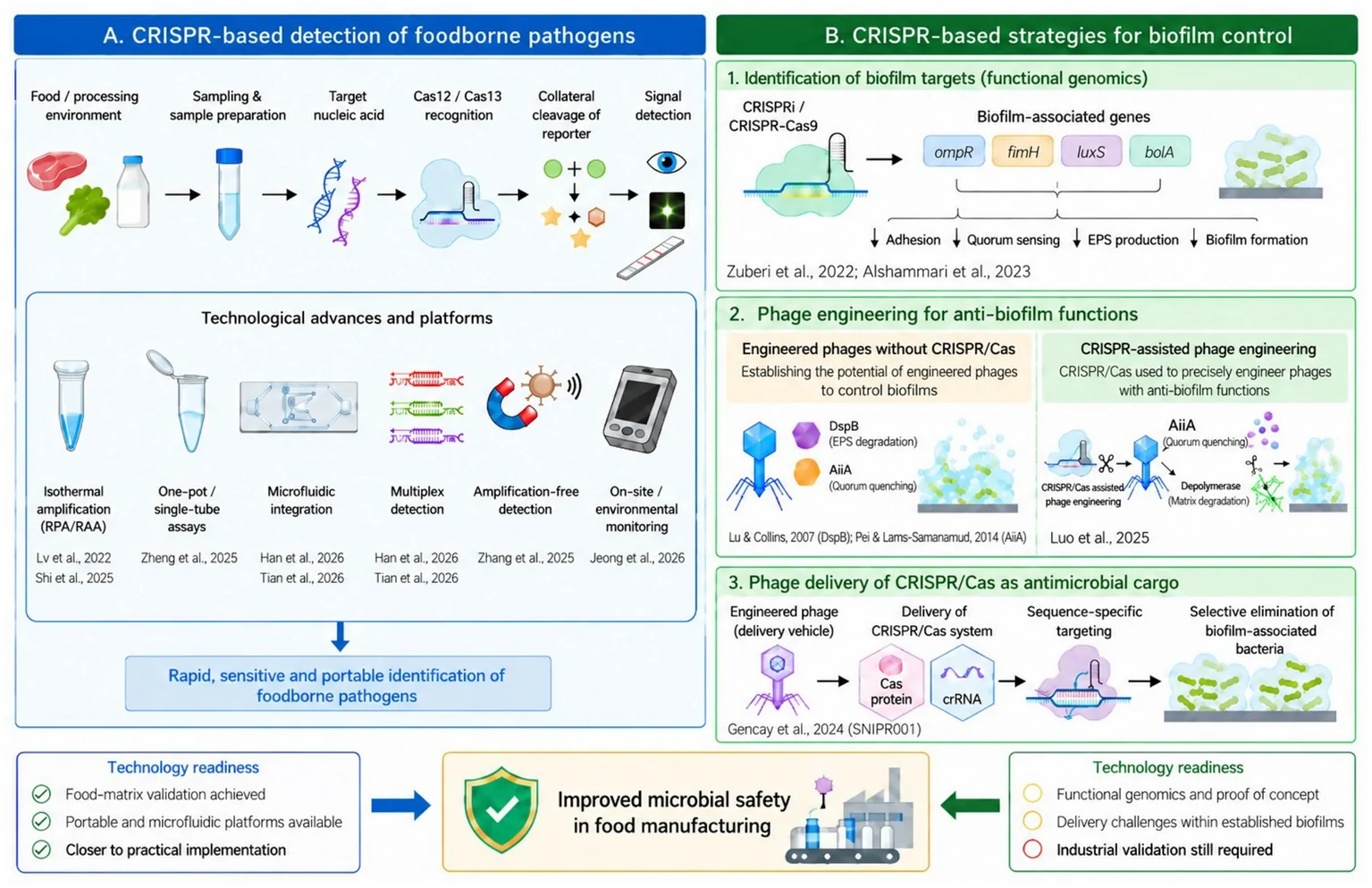
Conceptual schematic of CRISPR-based strategies for foodborne pathogen detection and biofilm control in food manufacturing. (A) CRISPR-based detection workflows and technological platforms (isothermal amplification, one-pot assays, microfluidics, amplification-free and on-site formats); (B) CRISPR-based approaches for biofilm control, ranging from functional genomics to CRISPR-assisted phage engineering and phage-delivered CRISPR antimicrobials. The lower panel highlights the difference in technology readiness: pathogen detection is closer to practical implementation, whereas biofilm control remains largely at the proof-of-concept stage. AiiA, autoinducer-inactivating lactonase; DspB, dispersin B; Cas, CRISPR-associated protein; CRISPR, clustered regularly interspaced short palindromic repeats; CRISPRi, CRISPR interference; crRNA, CRISPR RNA; EPS, extracellular polymeric substances; RAA, recombinase-aided amplification; RPA, recombinase polymerase amplification.

#### 3.5.2 CRISPR-based strategies for biofilm control

Compared with pathogen detection, CRISPR/Cas applications for direct biofilm control are considerably less mature. Biofilms are structured microbial communities embedded in an extracellular matrix that promotes surface attachment and enhances persistence under environmental stress and antimicrobial treatment. CRISPR-based genome editing and transcriptional repression offer powerful approaches for identifying genetic determinants of adhesion, quorum sensing, extracellular matrix production, and biofilm maturation (Noirot-Gros et al., 2019; Rahman et al., 2025). However, reducing biofilm formation by genetically modifying a laboratory strain differs from directly eliminating an established biofilm from a food-processing surface. CRISPR interference has been used to investigate regulatory pathways involved in *E. coli* biofilm development. Zuberi et al. (2022) used dCas9-mediated CRISPRi to suppress *ompR*, a component of the OmpR/EnvZ two-component regulatory system. Downregulation of *ompR* was associated with reduced *fimH* expression and decreased biofilm formation, as confirmed by quantitative and microscopic analyses. The study therefore identified OmpR-mediated regulation as a potential molecular target for biofilm intervention and demonstrated the utility of CRISPRi for reversible functional analysis of biofilm-associated pathways.

Direct genome editing provides further evidence for the role of specific genes in biofilm formation. Alshammari et al. (2023) used CRISPR/Cas9-mediated homology-directed repair to disrupt *luxS*, *fimH*, and *bolA* in *E. coli* ATCC 25922, targeting quorum sensing, adhesion, and biofilm regulation. Disrupting these genes reduced biofilm formation by about 75–84% and decreased extracellular polymeric substance production. The study also showed reduced adhesion in a catheter-associated biofilm model. Although this study was conducted in a medical rather than a food-processing context, it provides strong mechanistic evidence that CRISPR-based disruption of quorum-sensing and adhesion pathways can markedly alter the biofilm phenotype.

#### 3.5.3 Bacteriophage-based strategies and CRISPR-assisted phage engineering

A major obstacle to translating CRISPR-based biofilm control into food manufacturing is efficiently delivering CRISPR/Cas components to target bacterial cells within established biofilms (Gencay et al., 2024; Rahman et al., 2025). Genetically disrupting a biofilm-related gene in a laboratory strain before biofilm formation differs fundamentally from delivering an active CRISPR system to bacterial populations embedded in a mature extracellular matrix. Bacteriophages are a potential delivery platform for targeting bacteria within biofilms because of their natural host specificity and capacity to carry additional genetic or antimicrobial functions (Stout et al., 2017; Rahman et al., 2025; Juszczuk-Kubiak, 2024).

Engineered phages have long been shown to disrupt biofilms, even before the advent of CRISPR-based phage engineering. Lu and Collins (2007) engineered T7 to express dispersin B (DspB). This enzyme degrades a major component of the *E. coli* biofilm matrix, reducing biofilm-associated bacterial cells by approximately 4.5 logs. Pei and Lamas-Samanamud (2014) subsequently engineered T7 to express AiiA, an N-acylhomoserine lactonase that disrupts quorum sensing, reducing mixed-species *E. coli–Pseudomonas aeruginosa* biofilms by 74.9 and 65.9% after 4 and 8 h, respectively. Although these studies did not employ CRISPR/Cas, they established the concept of using engineered phages to combine bacterial targeting with additional anti-biofilm functions. More recently, CRISPR-Cas9 has been used directly as a phage-engineering tool to modify the *P. aeruginosa* phage PaGZ-1 to express either AiiA or a phage-derived depolymerase (Luo et al., 2025). Both engineered phages inhibited biofilm formation more than the wild-type phage, and the AiiA-expressing variant also showed activity against established biofilms. These findings demonstrate the potential of CRISPR/Cas-mediated phage engineering to enhance antibiofilm activity under experimental conditions, although the effectiveness of this approach on food-contact surfaces and in food-processing environments remains to be established.

Beyond phage genome engineering, CRISPR/Cas can also serve as sequence-specific antimicrobial cargo delivered to target bacteria by engineered phages. Gencay et al. (2024) incorporated antibacterial CRISPR–Cas systems into engineered phages targeting *E. coli*. The resulting CRISPR–Cas-armed phages remained active against biofilm-associated bacteria and reduced the emergence of phage-tolerant populations, with four complementary engineered phages ultimately combined to form SNIPR001. Although SNIPR001 was developed to therapeutically deplete intestinal *E. coli* rather than decontaminate food surfaces, it provides an important proof of concept for using CRISPR-equipped phages as programmable antimicrobials against biofilm-associated bacterial populations (Gencay et al., 2024). Studies using conventional, non-CRISPR-engineered bacteriophages directly on food-contact materials further support the potential relevance of phage-mediated approaches to food manufacturing. Brás et al. (2024) demonstrated that treatment with phage phT4A reduced *E. coli* biofilms by up to 5.5 log CFU/cm^2^ on plastic and approximately 4.0 log CFU/cm^2^ on stainless steel after 6 h. However, biofilm regrowth was observed after prolonged incubation. More recently, Kang et al. (2026) reported that the lytic phage CAU_ECP01 reduced preformed *E. coli* O157:H7 biofilms by 2.0 and 1.9 log CFU/cm^2^ on stainless steel and high-density polyethylene, respectively, and decreased bacterial contamination by 1.2 and 2.3 log CFU/g in inoculated beef and lettuce. Although these studies do not demonstrate CRISPR-mediated biofilm control, they provide translational evidence that phage-based bacterial targeting can be effective on food-contact surfaces and in food matrices, supporting further development of CRISPR-engineered phages specifically adapted to food-processing environments.

## 4 Technical challenges and limitations of CRISPR/Cas engineering in food-associated microorganisms

Despite rapid progress in CRISPR/Cas-based engineering of food-associated microorganisms, the transition from proof-of-concept genome editing to robust and industrially applicable strain development remains constrained by several technical barriers. Unlike well-established model organisms, many starter cultures, probiotic bacteria, and other food-relevant microorganisms are genetically recalcitrant and possess highly strain-specific physiological and genomic characteristics (Cui and Qu, 2024). Consequently, an editing strategy that performs efficiently in one strain may show substantially lower activity or fail completely in another. Major limitations include species- and strain-dependent editing efficiency, inefficient delivery of CRISPR components, restriction–modification (R–M) systems, Cas-associated toxicity, limited DNA-repair capacity, genetic instability, and the need to eliminate selectable markers and editing plasmids from the final strain (Cui and Qu, 2024; Xin et al., 2025). These constraints are particularly relevant to food biotechnology, where successful genome modification must ultimately be combined with genetic stability, technological performance, and compatibility with food-production conditions (Lv et al., 2023; Xin et al., 2025).

The major technical challenges and limitations of CRISPR/Cas-based genome engineering in food-associated microorganisms are summarized in Figure 4.

**Figure 4:**
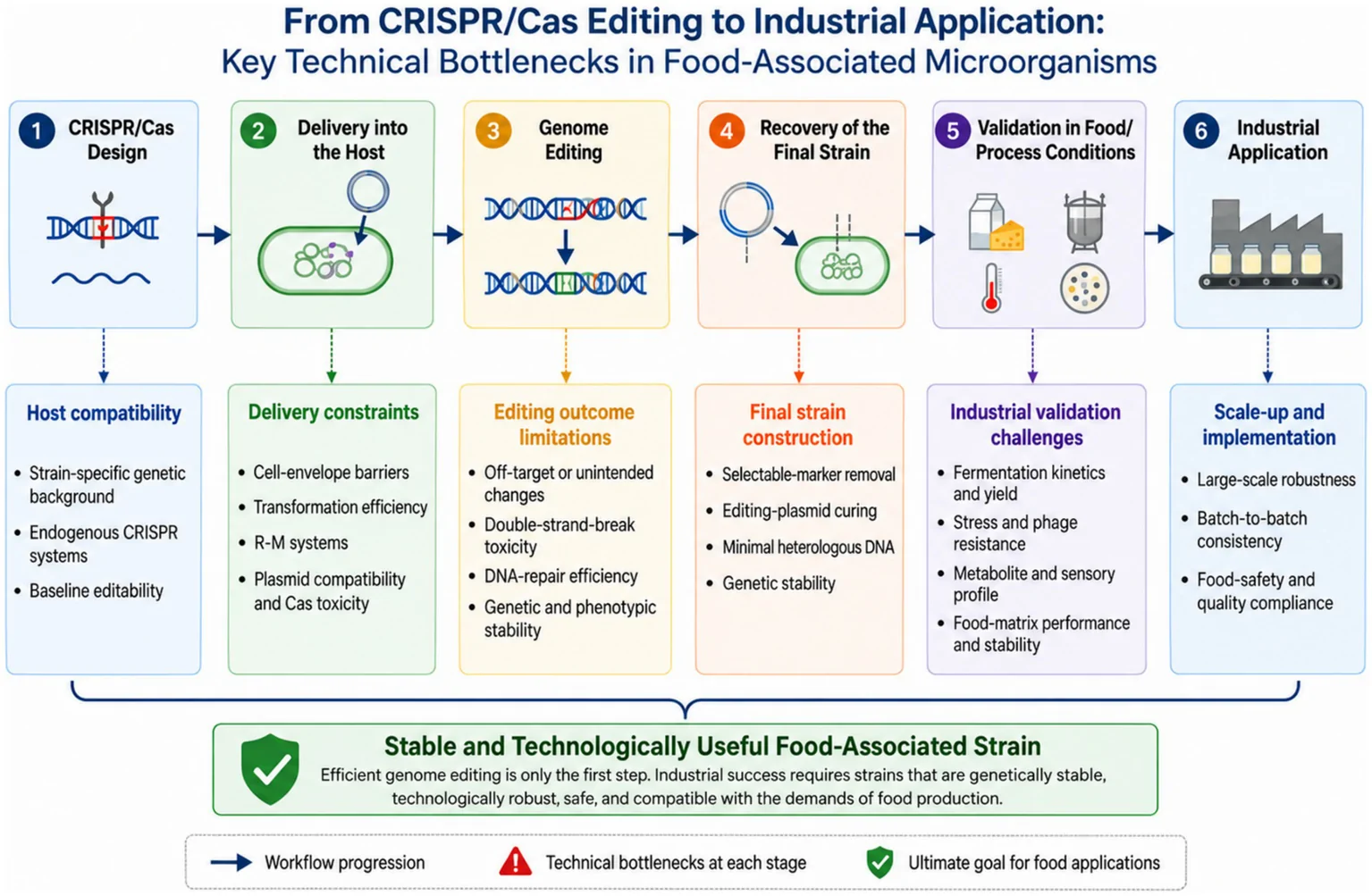
Conceptual schematic of the main stages and key technical bottlenecks in translating CRISPR/Cas editing into industrial applications involving food-associated microorganisms. The upper row shows the main workflow stages, while the lower row summarizes the major technical constraints at each stage. Cas, CRISPR-associated protein; CRISPR, clustered regularly interspaced short palindromic repeats; DNA, deoxyribonucleic acid; R–M, restriction–modification.

### 4.1 Species- and strain-dependent editing efficiency

CRISPR/Cas performance is strongly dependent on the recipient strain. Food-associated bacteria and yeasts differ in transformation efficiency, DNA repair, endogenous nuclease activity, plasmid compatibility, and susceptibility to Cas-mediated cleavage (Leenay et al., 2019; Rozanov et al., 2025). In *L. plantarum*, editing outcomes varied by strain and method even at the same locus, and some isolates contained unexpected genomic changes (Leenay et al., 2019). In *C. butyricum*, constitutive heterologous Cas9 expression caused toxicity and poor transformation, whereas repurposing the endogenous type I-B system enabled deletion of *spo0A* and *aldh* with efficiencies up to 100% (Zhou et al., 2021). Similar strain-to-strain variability, restrictive cell envelopes, limited validated genetic parts, and extensive restriction-modification systems constrain engineering in *Bifidobacterium* (Shin and Barrangou, 2026). Thus, platforms generally require host-specific optimization rather than direct transfer between strains. These studies show that the effectiveness of CRISPR/Cas engineering in food-associated microorganisms depends not only on the properties of the editing system itself but also on the genetic and physiological characteristics of the recipient host. Developing more broadly transferable editing platforms therefore remains an important objective; however, current evidence indicates that substantial host- and strain-specific optimization is still required.

### 4.2 Delivery of CRISPR/Cas components

Delivery of CRISPR/Cas components remains a major bottleneck in food-associated bacteria (Mougiakos et al., 2018; Plavec and Berlec, 2020; Li et al., 2024; Rozanov et al., 2025). Editing may require simultaneous introduction of Cas, guide RNA, donor DNA, and recombinase modules, while uptake and construct maintenance vary among hosts (Oh and van Pijkeren, 2014; Leenay et al., 2019). Electroporation is widely used in LAB and bifidobacteria but depends on cell-envelope properties, culture conditions, and incoming DNA. Optimisation increased transformation in *Bifidobacterium bifidum* BGN4 (Park et al., 2019) and raised efficiency in *B. animalis* AR668 to 7.2 × 10^5^ CFU/microgram DNA when combined with competence-gene expression and plasmid modification (Li et al., 2024). Host-compatible DNA methylation increased transformation in *Bifidobacterium adolescentis* by approximately five orders of magnitude and also improved transformation in *L. lactis* (Yasui et al., 2009). Consistently, bypassing restriction-modification barriers improved cytosine base editing in genetically recalcitrant bifidobacteria (Lin et al., 2025). Poor editing performance may therefore reflect loss of incoming constructs before genome modification rather than inadequate Cas activity alone.

One strategy to reduce the delivery burden is to exploit endogenous CRISPR/Cas systems, avoiding the need to introduce and express a heterologous Cas nuclease. In *P. acidilactici* LA412, Liu L. et al. (2021a) repurposed the endogenous type II-A CRISPR/Cas system for marker-free genome engineering, enabling targeted gene deletion, chromosomal integration, and point mutation. Similarly, in *C. butyricum*, constitutive expression of heterologous Cas9 caused substantial toxicity and very poor transformation efficiency, whereas repurposing the endogenous type I-B CRISPR/Cas machinery enabled efficient genome modification, including deletion of *spo0A* and *aldh* (Zhou et al., 2021). These examples illustrate how endogenous CRISPR/Cas systems can reduce the requirement for heterologous Cas expression and improve compatibility between the editing architecture and the recipient host.

Cas-mediated DNA cleavage represents an additional constraint because successful delivery of CRISPR/Cas components does not necessarily ensure recovery of edited cells. In many bacteria, Cas9-induced double-strand breaks can be lethal when DNA repair or homologous recombination is inefficient, making the balance between nuclease activity, donor-DNA availability, and cellular repair capacity critical for successful genome editing (Lv et al., 2023; Cui and Qu, 2024). This limitation has encouraged the development of editing architectures that reduce or avoid lethal double-strand cleavage. For example, Song et al. (2017) developed a Cas9D10A nickase-assisted system for genome editing in *Lacticaseibacillus casei*, while more recent base-editing approaches in *Bifidobacterium* have enabled targeted nucleotide modification without introducing double-strand DNA breaks (Lin et al., 2025). CRISPR interference provides another DSB-free strategy when modulating gene expression rather than permanently modifying the genome is sufficient.

### 4.3 Genetic integrity and marker-free genome engineering for food applications

Successful recovery of edited colonies does not necessarily guarantee the accuracy or genetic stability of the resulting strain. Unintended genomic changes can arise during genome editing and strain construction, particularly when Cas-mediated counterselection and recombination are used. In *L. plantarum*, Leenay et al. (2019) showed that editing outcomes varied across strains and editing strategies and identified unexpected genomic alterations in some edited isolates. These findings highlight the importance of verifying both the intended modification and the overall genomic integrity of edited strains rather than relying solely on apparent editing efficiency. Double-strand-break-free approaches may reduce some limitations associated with nuclease-mediated chromosome cleavage. For example, efficient cytosine base editing without double-strand DNA breaks has recently been demonstrated in *Bifidobacterium* (Lin et al., 2025). However, the absence of double-strand breaks does not eliminate the need to assess potential unintended sequence changes and phenotypic stability.

For microorganisms intended for food applications, another important consideration is the genetic composition of the final edited strain. Conventional genome-engineering workflows frequently rely on selectable markers, heterologous plasmids, or other auxiliary genetic elements that facilitate strain construction but are undesirable in the final production strain. Consequently, marker-free editing and efficient removal of editing constructs are key objectives in developing CRISPR platforms for food-associated microorganisms. Zhou et al. (2023) developed a food-grade CRISPR/Cas9–Red/ET system for *L. lactis* NZ9000 that achieved knockout efficiencies of up to 91% and enabled the recovery of edited strains without retained antibiotic-resistance markers or exogenous editing elements.

Removing editing plasmids offers another strategy to minimize the genetic footprint of genome engineering. Gu et al. (2022) repurposed the endogenous CRISPR/Cas9 system of *L. paracasei* and incorporated a controllable plasmid-targeting sgRNA module into an all-in-one editing vector, enabling efficient genome modification followed by plasmid curing with efficiencies exceeding 90%. Similarly, Garay-Novillo et al. (2024) developed pH-regulated CRISPR/Cas9 vectors for *Lactococcus cremoris* that combined controlled Cas9 expression with efficient plasmid loss without selection, thereby facilitating recovery of plasmid-free edited strains. Together, these approaches demonstrate how transient editing systems, endogenous CRISPR machinery, and easily curable vectors can reduce the persistence of auxiliary genetic elements in strains intended for food biotechnology. Importantly, marker-free or plasmid-free construction does not, in itself, establish the safety or regulatory acceptability of an edited microorganism for food use. The intended genomic modification, possible unintended changes, genetic and phenotypic stability, and the strain’s final technological properties must also be considered. Regulatory and biosafety implications are discussed separately in Section 5.

### 4.4 From laboratory editing to industrial performance

A major limitation in evaluating CRISPR engineering of food-associated microorganisms is that editing efficiency does not necessarily translate into technological performance. Although researchers have developed increasingly efficient CRISPR toolkits for food-related microorganisms, successful genome modification is only the first step toward obtaining strains suitable for technological applications (Lv et al., 2023; Xin et al., 2025). More broadly, recent analyses of genome editing in food systems have highlighted a persistent validation gap between molecular-level editing outcomes and phenotypic performance under food-relevant conditions (Sekerci Keles and Esen, 2026).

An intended genetic modification may affect multiple physiological and metabolic traits, including growth, substrate utilization, stress tolerance, metabolite balance, and interactions with other microorganisms (Liu L. et al., 2025). These effects may be negligible under laboratory conditions but become important during food fermentation, when microorganisms encounter dynamic changes in nutrient availability, pH, temperature, osmotic pressure, oxygen exposure, microbial competition, and other processing stresses (Derunets et al., 2024; Auchtung et al., 2025). This is particularly relevant to metabolic engineering, where redirecting metabolic flux toward a desired product may affect other traits required for robust fermentation performance (Lv et al., 2020; Liu L. et al., 2025). The importance of process-relevant validation is illustrated by studies evaluating CRISPR-engineered strains beyond standard laboratory media. In an industrial baker’s yeast, CRISPR/Cas9-mediated engineering was followed by testing in low- and high-sugar doughs, demonstrating improvements in leavening performance, osmotic and freeze tolerance, and selected bread properties (Cha et al., 2025). Similarly, CRISPR/Cas9-based metabolic engineering of *S. cerevisiae* for Chinese rice wine production was validated in brewing experiments, where the engineered strain reduced urea and ethyl carbamate concentrations by 92.0 and 58.5%, respectively, compared with the parental strain (Wu et al., 2020). These studies underscore the need to assess whether molecularly engineered traits remain beneficial in the intended technological environment.

Overall, the key challenge is moving from editing food-associated microorganisms to predictably engineering stable, technologically useful phenotypes. Future studies should therefore place greater emphasis on validating engineered strains under process-representative fermentation conditions and in representative food matrices, rather than relying predominantly on editing efficiency or laboratory-scale phenotypes. Such validation will be essential to translate CRISPR/Cas from an efficient microbial genome-editing technology into a reliable platform for precision engineering in food biotechnology.

## 5 Regulatory pathways, biosafety assessment, and consumer acceptance of CRISPR-engineered food microorganisms

Translating CRISPR/Cas-engineered microorganisms from laboratory research to food applications depends not only on technological performance but also on regulatory classification, biosafety assessment, and consumer acceptance. These issues are particularly complex because food-associated microorganisms may be used in fundamentally different ways: as production organisms that are removed or inactivated during processing, as viable starter cultures that persist in fermented foods, or as probiotic microorganisms intentionally consumed in a viable form. Consequently, both the nature of consumer exposure and the information required for safety assessment depend strongly on the intended use of the engineered strain.

The European Union, the United States, Canada, Brazil, and Argentina are discussed as representative examples of jurisdictions with differing regulatory approaches to genetically engineered microorganisms, illustrating variations in regulatory classification, risk assessment, and oversight of their applications in food biotechnology.

Within the European Union, the contained use of genetically modified microorganisms (GMMs) is governed by Directive 2009/41/EC, which defines a GMM as a microorganism whose genetic material has been altered in a way that does not occur naturally by mating and/or natural recombination, and which establishes measures to protect human health and the environment during contained use (European Parliament, Council of the European Union, 2009). For food applications, Regulation (EC) No 1829/2003 establishes the framework for genetically modified food and feed, including food containing, consisting of, or produced from genetically modified organisms (European Parliament, Council of the European Union, 2003a), while Regulation (EC) No 1830/2003 addresses traceability and labeling requirements for GMOs and products produced from GMOs (European Parliament, Council of the European Union, 2003b). Therefore, the regulatory status of a CRISPR-edited microorganism cannot be inferred solely from the precision of the editing procedure or the absence of a persistent editing plasmid. Marker-free or plasmid-free construction may simplify the molecular characterization of the final strain, but it should not be interpreted as automatically placing the microorganism outside the applicable regulatory framework. Where viable genetically modified microorganisms are deliberately released or placed on the market as or in products, Directive 2001/18/EC may also be relevant, subject to its relationship with applicable sector-specific legislation (European Parliament, Council of the European Union, 2001).

In the United States, federal statutes regulate biotechnology products within the policy framework established by the Coordinated Framework for the Regulation of Biotechnology. Regulatory responsibilities are divided primarily among the Food and Drug Administration (FDA), Environmental Protection Agency (EPA), and United States Department of Agriculture (USDA), depending on the nature, intended use, and potential risks of the product; consequently, a single product may fall within the jurisdiction of more than one agency (Executive Office of the President, 2017; USDA et al., 2023). For food applications, the FDA generally oversees food and food ingredient safety under the Federal Food, Drug, and Cosmetic Act. Substances produced by genetically modified microorganisms may require premarket approval as food additives unless their intended use is generally recognized as safe (GRAS) (FDA, 2026). Depending on the intended application, modified microorganisms may also fall under EPA oversight, for example as microbial pesticides or certain industrial microorganisms, or under USDA oversight where plant or animal health legislation applies.

In South America, Brazil regulates GMOs and their derivatives under the Biosafety Law (Presidency of the Republic Brazil, 2005), with the National Technical Commission on Biosafety (CTNBio) playing a central role in scientific risk assessment and regulatory decisions on GMO-related activities. Importantly, CTNBio Normative Resolution No. 16/2018 established a case-by-case procedure to determine whether products developed using precision breeding techniques fall within the GMO regulatory framework (CTNBio, 2018). Argentina similarly conducts case-by-case biosafety assessments through its national biotechnology framework and CONABIA, with current regulations covering genetically modified organisms intended for agricultural, agri-food, and agro-industrial applications (Ministerio de Economía, Argentina, 2026). These differences show that the regulatory pathway for CRISPR-engineered food microorganisms remains jurisdiction-dependent and depends not only on the modification itself but also on the intended use, exposure scenario, and characteristics of the final product.

Across these regulatory frameworks, biosafety assessment extends beyond confirmation of the intended genomic modification. For microorganisms used in food, relevant considerations include unequivocal strain identification, molecular characterization of the modification, genetic stability, potential unintended genomic or phenotypic effects, antimicrobial-resistance determinants, virulence- or toxin-associated traits, and metabolic changes that could affect food composition or safety. EFSA guidance for microorganisms used as feed additives or production organisms emphasizes whole-genome-sequence-based characterization, antimicrobial resistance, and toxigenicity and pathogenicity-related properties as central elements of microbial assessment (EFSA FEEDAP Panel, 2018; EFSA Scientific Committee, 2025). Although this guidance does not address CRISPR-edited food cultures specifically, it illustrates the broader shift toward strain-level genomic and phenotypic characterization in microbial risk assessment.

Health Canada provides a particularly useful microorganism-specific framework, with guidelines that explicitly address novel foods derived from genetically modified microorganisms. The assessment includes characterization of the derived strain; description and characterization of the genetic modification; history of the host and donor organisms; dietary exposure; nutritional and toxicological considerations; allergenicity; and microbiological characteristics (Health Canada, 2006). Importantly, the framework is comparative and considers both intended and unintended effects relative to a conventional counterpart with a history of safe use. It also recognizes that genetic modification may inadvertently alter pathogenicity, toxicity, allergenicity, physiological characteristics, or food composition. Exposure differs fundamentally between production strains absent from the final food and viable engineered cultures. Health Canada indicates that gastrointestinal viability and residence may not require evaluation when processing eliminates viable genetically modified microorganisms, whereas viable recombinant microorganisms may require assessment of gastrointestinal residence and effects on the microbiota (Health Canada, 2006). A production strain used to manufacture an ingredient therefore presents a different scenario from a viable starter culture or probiotic (EFSA GMO Panel, 2011; European Food Safety Authority, 2024). Assessing viable engineered microorganisms may need to address survival, persistence, microbiota interactions, metabolite production, and horizontal gene transfer, in addition to conventional strain-level characteristics (Sanders et al., 2010; Merenstein et al., 2023). Requirements should remain proportionate to the modification, intended function, and exposure. Editing specificity and removal of selectable markers do not replace phenotypic characterization or demonstrate safety (EFSA GMO Panel, 2011; European Food Safety Authority, 2024).

Consumer acceptance represents another important consideration for the industrial implementation of CRISPR/Cas technology. However, direct evidence concerning CRISPR-edited starter cultures, probiotics, or foods containing such microorganisms remains scarce. Most consumer surveys and choice experiments conducted to date have focused on gene-edited crops and plant-derived foods, including rice, apples, tomatoes, salad greens, wheat flour, and orange juice (Shew et al., 2018; Paudel et al., 2023; Deng et al., 2023; Deka et al., 2025). Consequently, consumer attitudes toward foods produced using gene-edited microorganisms, particularly products containing viable engineered strains, remain largely unexplored. Although not directly equivalent, evidence from precision-fermented foods indicates that acceptance depends on perceived benefits and risks, naturalness, trust, food-technology neophobia, and communication (Banovic and Grunert, 2023; Kühl et al., 2024; Hilgendorf et al., 2024). Framing precision fermentation as similar to traditional fermentation increased trust and acceptance, whereas explicitly emphasizing genetic modification modestly reduced willingness to try precision-fermentation-derived cheese (Banovic and Grunert, 2023; Kühl et al., 2024). Studies of Gouda alternatives similarly found safety, nutritional value, taste, perceived naturalness, and risk to be stronger determinants than broad societal benefits (Kilimann et al., 2026; Zeilinger and Riefler, 2026).

## 6 Discussion and future perspectives

The next stage in developing CRISPR/Cas technologies for food biotechnology will require a shift from demonstrating editing feasibility to establishing whether the resulting microorganisms perform stably and predictably under realistic food-production conditions (Börner et al., 2019; Xin et al., 2025). The reviewed studies show that CRISPR/Cas can generate technologically relevant microbial phenotypes, including improved stress or phage resistance, altered metabolite production, and modified probiotic properties. However, most studies have demonstrated these effects using single genetic targets under controlled laboratory conditions. Industrial strains must retain their engineered phenotypes during repeated cultivation, scale-up, fermentation, processing, and storage, where nutrient availability, pH, temperature, oxygen exposure, osmotic stress, and microbial interactions may vary considerably. Future studies should therefore investigate genotype-to-phenotype relationships under technologically relevant conditions and validate engineered strains directly in food matrices and production-like environments. Such evaluation is necessary to determine whether an experimentally measurable molecular effect confers a reproducible technological advantage.

A major challenge remains adapting and optimizing CRISPR/Cas platforms, including Cas9-based systems, for genetically diverse non-model food microorganisms. Although CRISPR/Cas9 is the most established genome-editing platform, its performance in food-associated bacteria does not transfer universally across species and strains. Editing efficiency may depend on transformation and delivery efficiency, endogenous defense mechanisms, Cas9-associated toxicity, the availability of suitable protospacer-adjacent motif (PAM) sequences, and the host’s DNA-repair capacity (Cui and Qu, 2024; Xin et al., 2025). Greater use of endogenous CRISPR/Cas systems, transient delivery of editing components, ribonucleoprotein-based approaches, and markerless strategies may reduce dependence on persistent plasmids and selectable markers (Börner et al., 2019; Cui and Qu, 2024). Nevertheless, the absence of persistent foreign DNA should not be equated with either regulatory exemption or demonstrated safety. CRISPR interference (CRISPRi), CRISPR activation (CRISPRa), base editing, and related approaches that modify gene expression or nucleotide sequences without conventional double-strand DNA cleavage may further expand the range of accessible phenotypes, particularly in strains for which nuclease-mediated editing is inefficient or highly toxic.

Integrating CRISPR/Cas with synthetic biology, systems biology, metabolic modeling, and multi-omics could shift the focus from single-gene modification to the rational engineering of complex microbial phenotypes. Genome-scale analyses can identify regulatory and metabolic nodes linked to fermentation performance, stress resistance, phage susceptibility, metabolite production, and interactions within microbial communities (Pan and Barrangou, 2020). CRISPR-based tools can then be used to validate these relationships and reprogram multiple targets through multiplex editing or coordinated transcriptional regulation. However, optimizing individual pathways alone is unlikely to improve complex traits. For example, redirecting metabolic flux toward a desirable compound may compromise growth, stress resistance, or fermentation kinetics. Future engineering strategies should therefore balance the intended phenotype with overall cellular fitness and process robustness. This approach may support the development of advanced starter cultures and next-generation or designer probiotics that combine technological stability with defined metabolic functions, including enhanced production of bioactive compounds and reduced formation of undesirable metabolites. It may also inform the design of production microorganisms adapted to particular food matrices, processing conditions, and precision-fermentation applications (Börner et al., 2019; Xin et al., 2025). Importantly, future applications should clearly distinguish food-grade starter cultures and probiotics intended for consumption as foods or dietary supplements from engineered live biotherapeutic products, which differ in their intended functions, exposure scenarios, safety assessment, and regulatory requirements.

The maturity and prospects of CRISPR-based applications vary substantially across food biotechnology. Genome editing has become an effective research tool for functional characterization and targeted strain improvement, yet comparatively few engineered food microorganisms have been validated at pilot or industrial scale. CRISPR-based diagnostics have advanced further toward practical food-safety applications, supported by increasingly rapid, multiplexed, and portable detection formats (Han et al., 2026; Jeong et al., 2026). Nevertheless, routine implementation still requires standardized preparation, control of matrix-associated inhibition, workflow integration, and validation across diverse food products. By comparison, engineered bacteriophages, sequence-specific CRISPR antimicrobials, and direct CRISPR-mediated control of established biofilms remain earlier in development. Their translation will depend on efficient delivery, activity within complex microbial communities and mature biofilms, avoidance of escape variants, and demonstrated efficacy under food-processing conditions.

These technological differences have significant biosafety and regulatory implications. Production microorganisms removed or inactivated during processing present a different exposure scenario from viable starter cultures or engineered probiotics that persist in the final food. Accordingly, assessment requirements should reflect the strain’s intended use, the nature of the modification, its genetic and phenotypic stability, and the extent of consumer and environmental exposure. High sequence specificity and removal of editing constructs cannot replace phenotypic characterization or evaluation of unintended biologically relevant effects. Regulatory clarity will therefore be essential to ensure consistent assessment of technically similar modifications while maintaining regulatory scrutiny proportionate to the associated exposure and risk.

Ultimately, the impact of CRISPR/Cas on the food industry will depend on whether molecular precision can be translated into microbial technologies that are stable, scalable, safe, economically viable, and compatible with food-production processes. Achieving this objective will require closer integration of genome engineering with phenotypic validation, process engineering, biosafety assessment, and regulatory analysis. Transparent communication about the purpose, benefits, and safety of CRISPR-engineered microorganisms will also be important, especially because consumer perceptions may differ between foods produced using an engineered production organism and those containing viable engineered cultures. If these challenges are addressed, CRISPR/Cas may evolve from a predominantly laboratory-based genome-engineering tool into a broader precision-microbiology platform for microbial strain development, targeted microbial control, and food-safety monitoring.

## 7 Literature search and selection

This narrative review was based on a targeted search of the scientific literature on CRISPR/Cas technologies and their applications in food microbial biotechnology. Relevant publications were identified through PubMed, Scopus, Web of Science, and Google Scholar, and the search was completed in September 2026. The search included combinations of terms related to CRISPR/Cas technologies, such as “CRISPR/Cas,” “Cas9,” “Cas12,” “Cas13,” “genome editing,” “gene knockout,” “base editing,” “prime editing,” “CRISPR interference,” and “microbial genome engineering”. Application-specific search terms included “food biotechnology,” “food microbiology,” “starter cultures,” “probiotics,” “metabolic engineering,” “bacteriophage resistance,” “foodborne pathogens,” “biofilm control,” and “CRISPR-based diagnostics”. Additional searches addressed “biosafety,” “genetically modified microorganisms”, “regulatory frameworks”, and “consumer acceptance”. Searches were further refined using microorganism-specific terms, including “Lactococcus”, “Lactobacillus”, “*Streptococcus thermophilus*”, and/or “*Saccharomyces cerevisiae*”.

The selected literature spanned 2004 to 2026, with particular emphasis on recent advances and earlier foundational studies that provided historical and technological context. Publications were selected for their relevance to the review’s scope, methodological significance, and experimental evidence supporting the reported applications. Priority was given to original research on food-associated microorganisms and to experimentally validated technological or functional outcomes. Studies conducted in biomedical or laboratory settings were also considered when they provided relevant mechanistic or technological insights; however, their applicability to food biotechnology was distinguished from direct validation in food systems. Relevant regulatory documents and scientific guidance were also included. Given the topic’s broad and interdisciplinary scope, a narrative rather than a systematic review approach was adopted (Ferrari, 2015).

## 8 Conclusion

CRISPR/Cas technologies offer a versatile platform for the precise engineering, functional characterization, and targeted control of food-associated microorganisms. Applications span starter and probiotic cultures, industrial metabolic engineering, pathogen control, biofilm research, and molecular diagnostics, though these areas vary widely in technological readiness. Efficient genome editing alone does not ensure industrial utility; it also requires genetic and phenotypic stability, robust performance in food matrices and under processing conditions, and maintenance of safety and product quality. Translation will further depend on reliable delivery, microorganism- and application-specific biosafety assessment, regulatory compliance, and consumer acceptance. Particular attention should be given to the nature of the modification, the intended function of the strain, the presence of viable engineered cells in the final product, and the level of consumer and environmental exposure. Addressing these requirements through transparent molecular and phenotypic characterization and validation under realistic food-use conditions will be essential for the responsible implementation of CRISPR/Cas in food biotechnology.
